# A *Helicobacter pylori* flagellar motor accessory is needed to maintain the barrier function of the outer membrane during flagellar rotation

**DOI:** 10.1371/journal.ppat.1012860

**Published:** 2025-01-10

**Authors:** Kyle Rosinke, Shoichi Tachiyama, Jan Mrásek, Jun Liu, Timothy R. Hoover

**Affiliations:** 1 Department of Microbiology, University of Georgia, Athens, Georgia, United States of America; 2 Department of Microbial Pathogenesis, Microbial Sciences Institute, Yale School of Medicine, New Haven, Connecticut, United States of America; 3 Institute of Bioinformatics, University of Georgia, Athens, Georgia, United States of America; Hudson Institute of Medical Research, AUSTRALIA

## Abstract

The *Helicobacter pylori* flagellar motor contains several accessory structures that are not found in the archetypal *Escherichia coli* and *Salmonella enterica* motors. *H*. *pylori hp0838* encodes a previously uncharacterized lipoprotein and is in an operon with *flgP*, which encodes a motor accessory protein. Deletion analysis of *hp0838* in *H*. *pylori* B128 showed that the gene is not required for motility in soft agar medium, but the mutant displayed a reduced growth rate and an increased sensitivity to bacitracin, which is an antibiotic that is normally excluded by the outer membrane. Introducing a plasmid-borne copy of *hp0838* into the *H*. *pylori* Δ*hp0838* mutant suppressed the fitness defect and antibiotic sensitivity of the strain. A variant of the Δ*hp0838* mutant containing a frameshift mutation in *pflA*, which resulted in paralyzed flagella, displayed wild-type growth rate and resistance to bacitracin, suggesting the fitness defect and antibiotic sensitivity of the Δ*hp0838* mutant are dependent on flagellar rotation. Comparative analysis of *in-situ* structures of the wild type and Δ*hp0838* mutant motors revealed the Δ*hp0838* mutant motor lacked a previously undescribed ring structure with 18-fold symmetry located near the outer membrane. Given its role in formation of the motor outer ring, HP0838 was designated FapH (flagellar accessory protein in *H**elicobacter pylori*) and the motor accessory formed the protein was named the FapH ring. Our data suggest that the FapH ring helps to preserve outer membrane barrier function during flagellar rotation. Given that FapH homologs are present in many members of the phylum Campylobacterota, they may have similar roles in protecting the outer membrane from damage due to flagellar rotation in these bacteria.

## Author summary

*Helicobacter pylori* causes a variety of gastric diseases in humans, including peptic ulcer disease, chronic gastritis, gastric cancer, and mucosa-associated lymphoid tissue lymphoma. Treatment of *H*. *pylori* infections is complicated by the need to combine multiple antibiotics and the rising occurrence of antibiotic resistance in clinical isolates, which underscores the need for new treatment options against this widespread and cancerogenic pathogen. The *H*. *pylori* flagellar motor shares core components with the *Escherichia coli* flagellum, but has evolved additional accessories that help the bacterium navigate the highly viscous gastric mucus layer. We report here on a previously uncharacterized ring-like *H*. *pylori* motor accessory associated with the outer membrane that is formed by the lipoprotein FapH. In the absence of FapH, *H*. *pylori* displays an increased sensitivity to bacitracin, an antibiotic that is normally excluded by the outer membrane. The increased antibiotic sensitivity of the *fapH* mutant is dependent on a functional flagellum, suggesting that the FapH ring protects the outer membrane from damage resulting from flagellar rotation. These findings identify the FapH ring as a potential target for novel therapeutics that may potentiate the activity of existing antibiotics in the treatment of *H*. *pylori* infections.

## Introduction

*Helicobacter pylori* is a Gram-negative bacterial pathogen of the phylum Campylobacterota that colonizes the gastric mucosa of approximately half of the human population worldwide [1]. *H*. *pylori* infection is the causative agent of chronic gastritis and peptic ulcer disease, as well as a major risk factor for gastric cancer [2–4]. The *H*. *pylori* flagellum is an essential virulence factor as flagellum-mediated motility is required for host colonization of the bacterium in animal models [5,6].

The bacterial flagellum consists of three major components: the basal body, hook, and filament. The basal body contains a rotary motor, which depending on the species uses energy from the proton motive force or a sodium ion gradient to generate torque [7–9]. The torque generated by the motor is transferred from the rotor to the filament via the rod and hook. The filament is a rigid helical structure that acts as a propeller to push the cell forward as the filament rotates. Conserved core structures of the basal body include a flagellar type III secretion system (fT3SS) that transports axial components (e.g., rod, hook, and filament proteins) across the cell membrane, C-ring (rotor and switch complex), MS-ring (rotor component), torque generating MotA/MotB or PomA/PomB stator units, P- and L-rings (bushings), and rod (driveshaft) [10,11]. The *H*. *pylori* motor, the largest bacterial flagellar motor described to date, accommodates 18 stator units that produce an estimated 3,600 pN·nm of torque [12]. In addition to the conserved core motor structures, high-resolution *in-situ* structures of the motors of *H*. *pylori* and other bacteria determined by cryo-electron tomography (cryo-ET) and subtomogram averaging reveal structures that are not present in the archetypal *Escherichia coli* and *Salmonella enterica* motors [10,13–15]. Functions for the motor accessories are largely unknown, but possible roles include recruitment and retention of stator units in the motor, stabilization of stator-rotor interactions, and protection of the cell envelope from the high torque generated by the motor [10,13–15].

The flagellar motor is anchored within the cell envelope and traverses the outer membrane (OM) in all diderms with the exception of the spirochetes. Depending on the species, the flagellar motor can rotate from hundreds to over 1,000 revolutions per second [16], rotational speeds that are comparable to those of a household blender. The bacterial cell body counterrotates to the direction of the flagellar motor rotation [16]. The rotation of the flagellum and counterrotation of the cell body has the potential to exert stress on the OM. The OM is an asymmetrical lipid bilayer with an outer leaflet populated by lipopolysaccharide (LPS) or lipooligosaccharide and an inner leaflet composed of glycerophospholipids (GPLs). The lipid asymmetry is important for OM barrier function and the intrusion of GPLs into the outer leaflet increases the permeability of the OM to harmful compounds that are normally excluded by the OM such as certain antibiotics and bile salts [17]. We hypothesize that in the absence of protective mechanisms the stress exerted on the OM by flagellar rotation facilitates the flipping of GPLs from the inner leaflet to the outer leaflet of the OM and thereby compromise OM barrier function.

In the *S*. *enterica* motor, the flagellar LP-ring bushing complex aligns and balances the flagellar rod as it rotates and is formed by multiple copies of FlgH and FlgI, which form the L-ring and P-ring, respectively [18,19]. The L-ring forms a ring in the OM fixed by interactions with FlgH and the lipid A moiety of LPS, and this ring allows the flagellar rod to pass through the OM [18]. *S*. *enterica* FlgH is a lipoprotein and the diacylglycerol attached to the side chain of the N-terminal cysteine residue of the protein aligns laterally to the L-ring and packs against hydrophobic amino acid residues of the protein. Rather than forming a typical membrane bilayer, the LPS layer and lipid moieties of FlgH form a hydrophobic band that encircles the L-ring [18]. The atypical membrane structure surrounding the L-ring may help to preserve OM barrier function by preventing the entry of GPLs into the outer leaflet of the OM during flagellar rotation.

The architecture of the *H*. *pylori* L-ring differs from that of *S*. *enterica*. *H*. *pylori* FlgH is not a lipoprotein, and the L-ring in the *H*. *pylori* motor is located below the OM rather than embedded in the OM [15]. Thus, if the *S*. *enterica* L-ring has a role in protecting the OM from flagellum-mediated damage, the *H*. *pylori* L-ring does not appear to be positioned for such a function. The *H*. *pylori* flagellum also differs from the *S*. *enterica* flagellum in that the filament of *H*. *pylori* flagellum is surrounded by a membranous sheath that is contiguous with the OM. The flagellar sheath is a feature that *H*. *pylori* shares with many other bacteria, including most *Helicobacter* and *Vibrio* species [20]. Fuerst proposed two models for the behavior of the sheath during flagellar rotation [21]. In one model, the sheath rotates with the filament, which requires that the sheath be rigid and the junction of the sheath and OM to be fluid. The other model proposes the filament rotates freely within a wave-propagating sheath. This second model requires the sheath to be flexible enough to allow for distortions caused by the rotating filament but rigid enough to prevent the sheath from detaching from the cell. Regardless of which model most accurately describes the behavior of the sheath as the filament rotates, rotation of sheathed flagella is a significant generator of outer membrane vesicles in *Vibrio* species [22,23], suggesting that flagellar rotation does indeed exert stress on the OM in bacteria with sheathed flagella.

The *H*. *pylori* flagellar motor has accessories associated with the *H*. *pylori* L-ring that may help to preserve OM barrier function as the flagella rotate. We provide evidence here that a ring-like motor accessory associated with the OM in *H*. *pylori* is formed by a lipoprotein that we designate FapH (flagellum-associated protein in *H**elicobacter pylori*). Deletion of *fapH* in *H*. *pylori* B128 resulted in increased sensitivity to bacitracin, an antibiotic that is normally excluded by the OM. The antibiotic sensitivity of the Δ*fapH* mutant was suppressed by introducing a copy of *fapH* on a shuttle vector into the strain. In addition, the antibiotic sensitivity of the Δ*fapH* mutant was dependent on functional flagella as a *H*. *pylori* Δ*fapH* mutant with paralyzed flagella due to a mutation in *pflA* displayed wild-type resistance to bacitracin. Taken together, these findings suggest that in the absence of the FapH ring, OM barrier function is compromised by rotation of the flagella. The breakdown in OM barrier function presumably results from the intrusion of GPLs into the outer leaflet of the OM, making the OM more permeable to bacitracin. FapH homologs are present in several other Campylobacterota, and these *fapH* homologs may function in protecting the OM from damage due to flagellar rotation in these bacteria.

## Results

### *fapH* (*hp0838*) is conserved in *Helicobacter* species and linked to *flgP*

**Fig 1:**
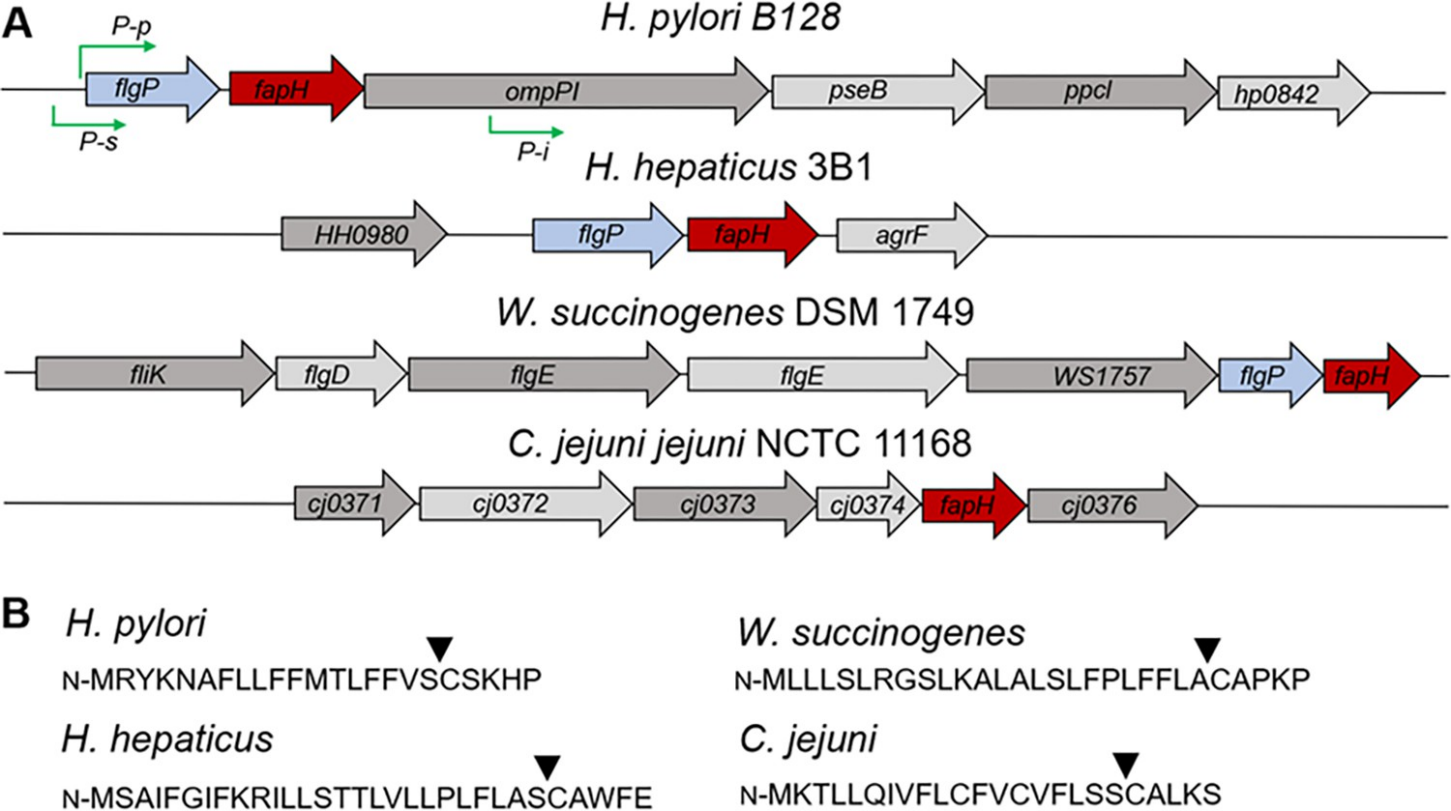
Organization of operons containing *fapH* in various members of Campylobacterota and the predicted lipoprotein signal peptides for the FapH homologs. (**A**) In many genomes, *fapH* is located immediately downstream of *flgP*. Approximate locations of predicted promoters for the operon containing *fapH* in *H*. *pylori* B128 are indicated (green arrows), which is based on a transcriptional start site database determined from the *H*. *pylori* 26695 transcriptome [63]. *P-p*: primary promoter, *P-s*: secondary promoter, *P-i*: internal promoter. (**B**) Predicted lipoprotein signal peptides of the FapH homologs. Amino acids sequences of the FapH homologs were analyzed for signal peptides and their cleavage sites using the SignalP-6.0 server (https://services.healthtech.dtu.dk/service.php?SignalP) [64]. Locations of the cleavage sites in the FapH homologs are indicated by the arrow.

*H*. *pylori* HP0838 (FapH) is a predicted lipoprotein encoded by a gene immediately downstream of *flgP*, which encodes a flagellar motor accessory protein that forms the basal disk in *Campylobacter jejuni* and *Aliivibrio fischeri* [13]. In the genomes of other *Helicobacter* species that we examined, a *fapH* homolog is located immediately downstream of a *flgP* homolog (**Fig 1 and S1 Table**). Further analysis of FapH using the Position-Specific Iterative (PSI)-BLAST tool identified FapH homologs in other members of the phylum Campylobacterota (**S1 Table**). In contrast to the synteny of *fapH* and *flgP* in *Helicobacter* species, however, *fapH* and *flgP* only display synteny in *Wolinella succinogenes* for the other Campylobacterota that we examined (**Fig 1 and S1 Table**). Notably, the *W*. *succinogenes fapH* homolog is in an operon with six other genes known or predicted to be involved in flagellum assembly (**Fig 1**), suggesting a role for *fapH* in flagellar function. As with *H*. *pylori* FapH, all of the FapH homologs that were examined have predicted lipoprotein signal peptides (**S1 Table**). Given the synteny between *flgP* and *fapH* in *Helicobacter* species and *W*. *succinogenes*, close association of *fapH* with several flagellar genes in *W*. *succinogenes*, and the predicted periplasmic location of FapH as a lipoprotein, we hypothesized that FapH is a flagellar motor accessory protein.

In examining the FlgP homologs of various *Helicobacter* species, we noted that many of the FlgP homologs possess predicted lipoprotein signal peptides, while others have signal peptides that are cleaved by signal peptidase I and are not predicted lipoproteins (**S1 Table**). This differentiation followed a morphological distinction as *Helicobacter* species that have sheathed flagella (FS^+^ species) typically possessed a FlgP homolog predicted to be a lipoprotein, while the FlgP homologs in *Helicobacter* species with sheath-less flagella (FS^-^ species) lacked a predicted lipoprotein signal peptide. Two FlgP homologs in FS^+^ *Helicobacter* species that we examined were not predicted to have signal peptides, but this may be due to incorrect assignments for the translational start sites as selecting alternative upstream translational start sites resulted in protein sequences with predicted lipoprotein signal peptides. The relationship between the type of signal peptide and sheathed/unsheathed flagella does not apply to all members of Campylobacterota as some species (e.g., *C*. *jejuni* and *Nautilia profundicola*) have FlgP homologs that are predicted lipoproteins but possess unsheathed flagella (**S1 Table**).

### FapH is not essential for motility in *H*. *pylori*

**Fig 2:**
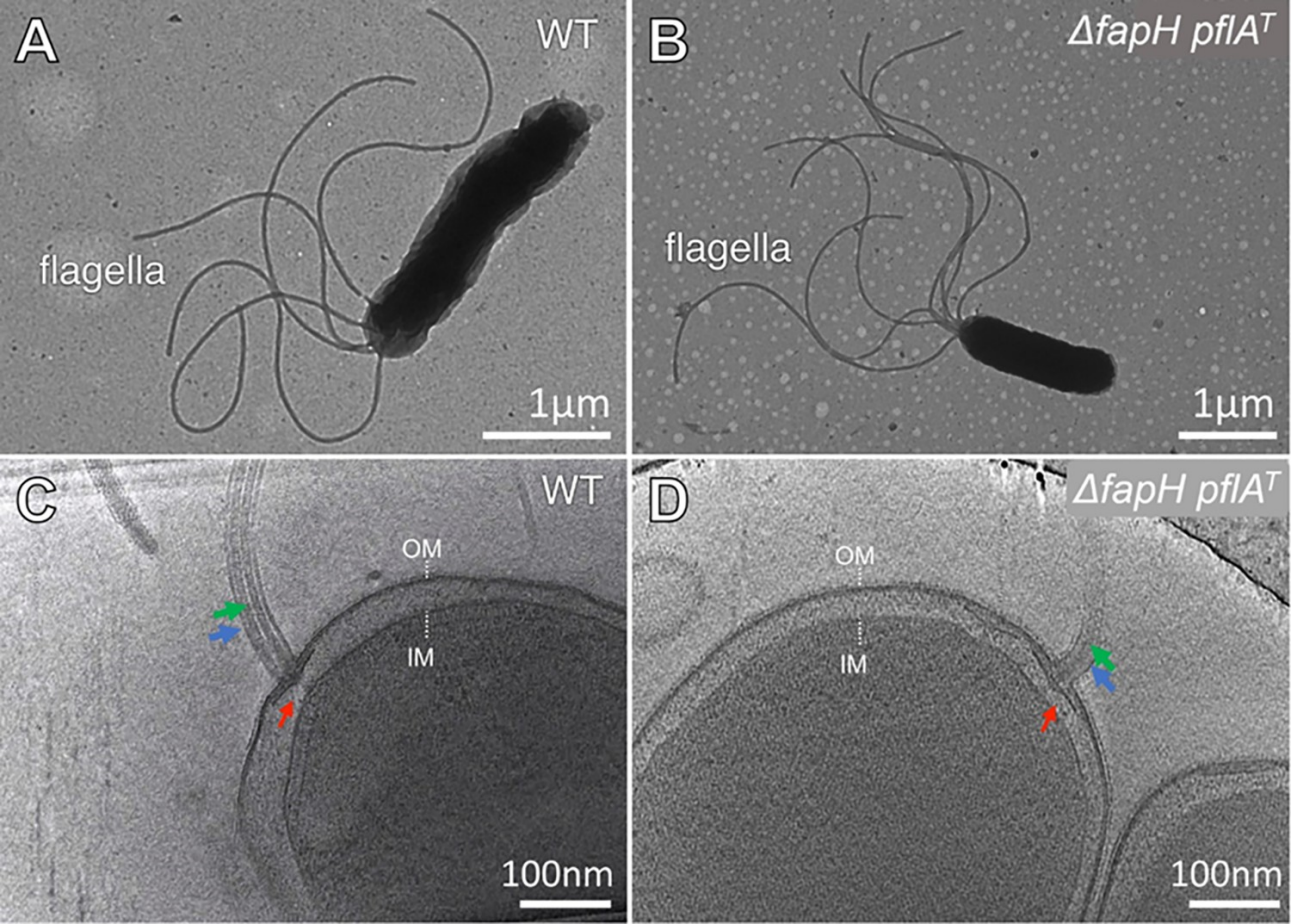
Electron micrographs of *H*. *pylori* B128 wild type and Δ*fapH pflA*^T^ strain. TEM images of representative cells of *H*. *pylori* B128 wild type (**A**) and Δ*fapH pflA*^T^ mutant (**B**). Section of cryo-ET images of *H*. *pylori* B128 wild type (**C**) and Δ*fapH pflA*^T^ mutant cells (**D**). In panels **C** and **D**, flagellar basal bodies are indicated by red arrows, flagellar sheath is indicated by the blue arrows, and flagellar filaments are indicated by the green arrows. OM–outer membrane; IM–inner membrane.

**Fig 3:**
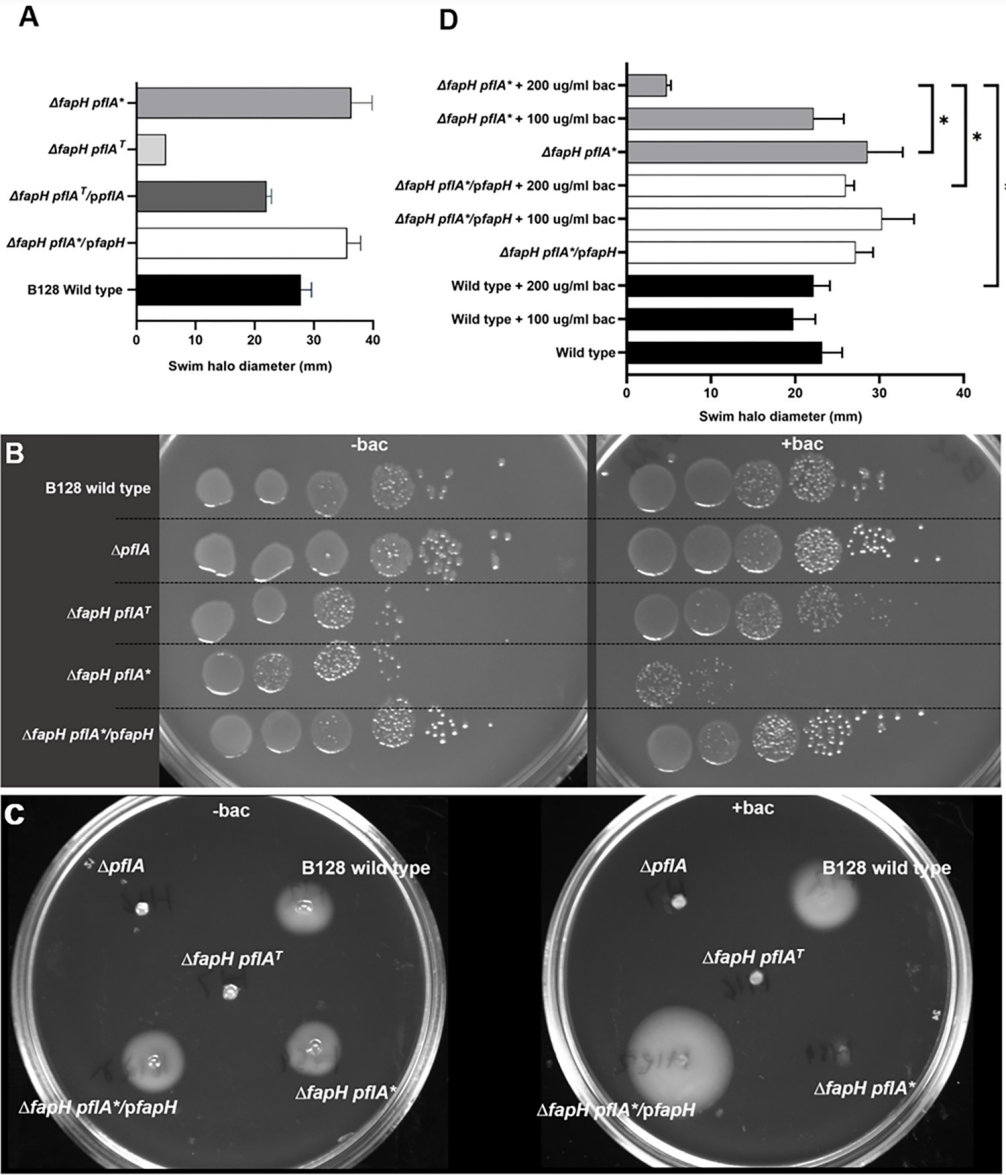
Motility of *H*. *pylori* B128 wild type and Δ*fapH* mutants and their sensitivity to bacitracin. (**A**) Motilities of wild type, Δ*fapH pflA*^T^, ΔfapH pflA*,Δ*fapH pflA*^T^*/*p*pflA*, and ΔfapH pflA*/p*fapH* were assessed by stab inoculating the strains in soft agar medium and then measuring the diameters of the resulting swim halos following a 7-d incubation period. Bars indicate mean values for the diameters of the swim halos and the error bars indicate the standard error of the mean (SEM). At least 4 replicates were used in motility assays. (**B**) Efficiency of plating assays were done with *H*. *pylori* strains (indicated in **A**) on TSA (-bac) or TSA supplemented with 200 μg/ml bacitracin (+bac). Cells from freshly grown cultures of the strains were resuspended in tryptic soy broth to the same cell densities (OD_600_). Ten-fold serial dilutions of the resuspensions (10^0^ to 10^−5^) were then spotted onto the media, and the cultures were incubated for 7 d. The Δ*pflA* mutant was included to demonstrate that disrupting *pflA* was not responsible for the increased sensitivity to bacitracin. (**C**) Growth of *H*. *pylori* strains in soft agar medium in the absence (-bac) and presence of 200 μg/ml bacitracin (+bac). (**D**) *H*. *pylori* B128 wild type, ΔfapH pflA*, and ΔfapH pflA*/p*fapH* were stab inoculated into soft agar medium that contained 0, 100, or 200 μg/ml bacitracin and the diameters of the resulting swim halos were measured following a 7-d incubation period. Bars indicate mean values for swim halo diameters and the error bars indicate the SEM. The asterisk indicates statistically significant differences in swim halo diameters as determined using a two-sample *t* test (*p*-value <0.0001). At least 3 replicates were done for each sample.

To determine if FapH plays a role in flagellar biosynthesis or function, *fapH* was deleted in *H*. *pylori* B128 and the flagellation and motility of the resulting mutant was assessed. Examination of the Δ*fapH* mutant by transmission electron microscopy (TEM) and cryo-ET revealed the cells were indistinguishable from wild type with regard to the flagellation pattern (**Fig 2).** Examples of cryo-ET images showing the flagellar sheath are presented in **Fig 2C and 2D**. The flagellar sheaths of the Δ*fapH* mutant appeared to be intact as evidenced by the uniform thickness of the flagella of the cells used for flagellar counts (n = 100), suggesting that FapH is not required for sheath biosynthesis. The *H*. *pylori* B128 Δ*fapH* mutant was impaired in its motility in soft agar medium as it generally produced no discernible swim halo following a 7-d incubation (**Fig 3A**). Migration of bacterial cells from the point of inoculation in soft agar medium involves both the ability to swim and chemotaxis. In our hands, a chemotaxis deficient *H*. *pylori* B128 Δ*cheA* mutant forms a small, but readily discernible, swim halo following a 7-d incubation [24]. Thus, the failure of the Δ*fapH* mutant to form a swim halo was not due to loss of chemotaxis, but instead resulted from the inability of the mutant to swim in the soft agar medium.

Small swim halos were sometimes observed on soft agar medium plates inoculated with the Δ*fapH* mutant, suggesting that motile suppressors of the Δ*fapH* mutant were arising. To enrich for motile variants of the Δ*fapH* mutant, cells from the edge of the small swim halos were used to inoculate fresh soft agar plates. Over the course of several passages in soft agar medium the average swim halo diameter of the Δ*fapH* mutant increased from ~5 mm to ~35 mm, and clonal isolates from the cells at the edges of these swim halos were obtained.

Whole genome sequencing (WGS) of the original Δ*fapH* mutant revealed an insertion of a guanosine at position 1429 in *pflA* (paralyzed flagellar protein A) (**S2 Table**). PflA is required for motility in *C*. *jejuni* and *H*. *pylori* [25,26]. *In-situ* structures of the *C*. *jejuni* motor generated by cryo-ET and single particle cryo-electron microscopy (cryo-EM) suggest PflA forms a set of spoke-like structures that are closely associated with the stator units [13,27]. Stator units are not visible in the *in-situ* structure for the motor of the *C*. *jejuni* Δ*pflA* mutant, suggesting that PflA is required for the recruitment and/or retention of the stator units within the motor [13]. The insertion in *pflA* shifted the reading frame and introduced a stop codon four codons downstream of the insertion, which is predicted to result in expression of a truncated PflA that is shortened by 325 amino acid residues. We hereafter refer to the original, non-motile Δ*fapH* mutant as the Δ*fapH pflA*^T^ strain. Introducing *pflA* on a shuttle vector into the Δ*fapH pflA*^T^ strain (hereafter designated as Δ*fapH pflA*^T^/p*pflA*) restored motility in soft agar medium to near wild-type levels (**Fig 3A**), indicating that the frameshift mutation in *pflA* was responsible for the loss of motility in the Δ*fapH pflA*^T^ strain. Moreover, these data indicated that FapH is not essential for motility in soft agar medium.

WGS analysis of a Δ*fapH* motile variant that produced a robust swim halo revealed a deletion of a cytosine at nucleotide position 1393 in *pflA* (**S2 Table**), which restored the reading frame following the insertion of the guanosine at position 1429 and allowed for the expression of a full-length PflA variant in which the amino acid sequence from Leu-465 to Ile-476 (L^465^FSMEGNTQEKI^476^) is altered to F^465^FPWRGTRKKKS^476^. PflA monomer structure predicted by AlphaFold 2 [28] possesses a β-sandwich domain near the N-terminus and a tetratricopeptide repeat (TPR) domain that comprises most of the remainder of the protein (**S1A Fig**). The insertion in Δ*fapH pflA*^T^ creates a truncation in the tetratricopeptide repeat region of PflA (**S1B Fig**). The altered sequence in PflA is primarily within a loop between α-helices 16 and 17 in the TPR domain, and the predicted tertiary structure of the PflA variant (hereafter referred to as PflA*) is essentially identical to that of wild-type PflA (**S1A and S1C Fig**). Given that the isolate expressing PflA* was highly motile and the amino acid substitutions did not impact the predicted tertiary structure of PflA, we proceeded to analyze further the motile isolate, hereafter referred to as the ΔfapH pflA* strain.

### The ΔfapH pflA* strain has reduced fitness

Although our data indicated that *fapH* is not required for *H*. *pylori* motility, we wished to determine if loss of *fapH* resulted in other phenotypic traits. Examination of the ΔfapH pflA* strain by TEM indicated that it was indistinguishable from wild type and the parental Δ*fapH pflA*^T^ strain in the number of flagella per cell (**S2A Fig**). Unexpectedly, the mean length of the flagellar filaments in the ΔfapH pflA* strain was slightly, but significantly, longer than those of the Δ*fapH* pflA^T^ strain and wild type (**S2B Fig**).

To determine if loss of FapH impacted the growth rate of *H*. *pylori*, we compared the growth rates of wild type, the non-motile Δ*fapH pflA*^T^ strain, and the motile ΔfapH pflA* strain. Growth rates for cultures of wild type and Δ*fapH pflA*^T^ strain were similar (mean doubling time was ~6 h; **S2C Fig**). By contrast, the growth rate of the ΔfapH pflA* strain was significantly slower with a mean doubling time of ~13 h (**S2C Fig**). To determine if deletion of *fapH* was responsible for the slower growth rate of the ΔfapH pflA* strain, *fapH* was introduced into the strain on the shuttle vector pHel3 to generate the strain that we hereafter refer to as ΔfapH pflA*/p*fapH*. Strain ΔfapH pflA*/p*fapH* had a growth rate similar to that of wild type and the Δ*fapH pflA*^T^ strain (**S2C Fig**). Taken together, these data suggest the loss of FapH results in a motility-dependent reduction of fitness in *H*. *pylori*.

### Strain ΔfapH pflA*, but not Δ*fapH pflA*^T^, has increased sensitivity to bacitracin

The LPS in the outer leaflet of the OM forms an effective barrier to hydrophobic antibiotics and other noxious chemicals, and increased sensitivity to these compounds is often indicative of the intrusion of GPLs into the outer leaflet of the OM [17]. Bacitracin is a cyclic polypeptide antibiotic that interferes with peptidoglycan biosynthesis by binding undecaprenyl-pyrophosphate to disrupt the undecaprenyl-phosphate cycle and thereby inhibit synthesis of the peptidoglycan precursor molecule lipid II [29]. In an efficiency of plating assay, the motile ΔfapH pflA* strain displayed increased sensitivity to bacitracin compared to wild type, the non-motile Δ*fapH pflA*^T^ strain, the motile ΔfapH pflA*/p*fapH* strain, and a Δ*pflA* mutant (**Fig 3B**). These data demonstrate that loss of FapH in *H*. *pylori* results in increased sensitivity to bacitracin and the antibiotic sensitivity is dependent on a functional flagellum. Moreover, these data show that disrupting *pflA* has no discernible effect on bacitracin sensitivity.

Sensitivity of the *H*. *pylori* strains to bacitracin was examined further under conditions where motility was assessed simultaneously by inoculating the strains in soft agar medium that contained bacitracin. *H*. *pylori* strains were inoculated into soft agar medium that contained 0, 100, or 200 μg/ml bacitracin and the subsequent swim halos were measured. Consistent with the results from the efficiency of plating assays, the ΔfapH plfA* strain displayed increased sensitivity to bacitracin as the swim halo diameter of the strain was markedly decreased in the presence of the antibiotic at a concentration of 200 μg/ml, while swim halo formation by wild type was unaffected (**Fig 3C and 3D**). Although the Δ*fapH pflA*^T^ strain did not form a swim halo since its flagella are paralyzed, colony growth of the strain in the soft agar medium containing bacitracin was dense indicating that the strain was resistant to bacitracin under the assay conditions (**Fig 3C**). As observed in the efficiency of plating assay (**Fig 3B**), the fapH pflA*/p*fapH* strain was resistant to bacitracin in the motility-based assay indicating that the loss of FapH was responsible for the antibiotic sensitivity of the ΔfapH pflA* strain (**Fig 3C and 3D**).

To address the possibility that PflA* is required for the antibiotic sensitivity of the ΔfapH pflA* strain, we generated additional Δ*fapH* mutants in *H*. *pylori* B128 and examined the sensitivity of the resulting strains to bacitracin. The resulting Δ*fapH* mutants were motile and sensitive to bacitracin (**S3 Fig**). WGS of the strains indicated that they did not have secondary mutations in any known flagellar gene, including *pflA* (**S2 Table**). These findings indicate that the sensitivity of *H*. *pylori* to bacitracin upon disruption of *fapH* is not dependent on the PflA variant PflA*.

### FapH forms a flagellar motor accessory associated with the OM

Given the sensitivity of the ΔfapH pflA* and Δ*fapH* strains to bacitracin, which is normally excluded by the OM, we hypothesized that FapH is required to form a motor accessory that helps to preserve OM barrier function during flagellar rotation. To address this hypothesis, we compared *in-situ* structures of the motors of wild type *H*. *pylori* B128, Δ*fapH plfA*^T^, and ΔfapH pflA*, which were determined by cryo-electron-ET and subsequent subtomogram averaging. The motor of Δ*fapH pflA*^T^ contained many of the core components and accessories that were present in the wild-type motor, but lacked many of the periplasmic components of the motor (**S4A and S4B Fig**). Since the periplasmic components are missing in the *in-situ* structure for the motor of the *C*. *jejuni* Δ*pflA* mutant [13], we infer that the truncation of PflA in Δ*fapH pflA*^T^ was responsible for the motor assembly and motility defects in the strain.

**Fig 4:**
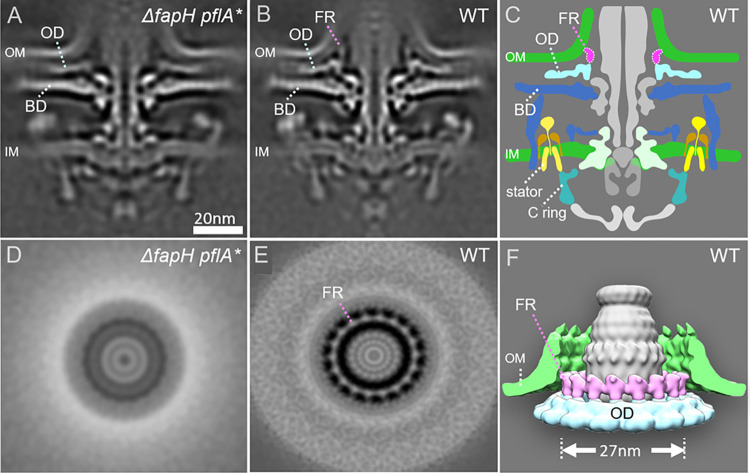
Architecture of the FapH ring in the *H*. *pylori* motor. (**A and B**) *In-situ* structures of the flagellar motors from ΔfapH pflA* and wild-type cells were determined using subtomogram averaging, respectively. Medial slices through the motor structures indicate the ΔfapH pflA* motor is composed of all motor components, except globular densities in the outer membrane region. (**C**) A model showing the location of the FapH ring and outer disk structures. (**D**) Cross section through the globular densities in the ΔfapH pflA* motor. (**E**) Cross section through the globular densities in the wild-type motor. (**F**) 3D model of the FapH ring adjacent to the outer membrane. FR–FapH ring, OD–outer disk, BD–basal disk, OM–outer membrane, IM–inner membrane.

Comparing the *in-situ* structures of the motors of wild type and ΔfapH pflA* revealed that the motors of the two strains were identical with the exception of a globular density near the junction of the OM and flagellar sheath that was missing in the motor of the ΔfapH pflA* strain (**Fig 4A–4C)**. Perpendicular cross-sectional imaging revealed that the globular densities form a ring-like structure with 18-fold symmetry that is absent in the ΔfapH pflA* motor (**Fig 4D and 4E**), and we designate the structure as the FapH ring. The FapH ring subunits are anchored on a motor accessory that we refer to as the outer disk and extend towards the OM (**Fig 4F**).

The tertiary structure of FapH predicted by Alphafold 2 [28] indicates α-helices near the N-terminus and at the C-terminus that are separated by a domain comprised of two β-sheets linked by three short α-helices (**S5A Fig**). The C-terminal α-helix of FapH is dominated by acidic amino acid residues, while the N-terminal α-helix and surface of the β-sheet domain contain several basic amino acid residues (**S5B Fig**). The predicted tertiary structure of FapH fits well into the density of the FapH ring subunits (**S5C Fig**), suggesting that each subunit represents a FapH monomer. From the modeling of the FapH tertiary structure on the FapH ring subunits, the β-sheet domain of FapH contacts the outer disk, while the N-terminal and C-terminal α-helices are distal to the outer disk and located at the top of the FapH ring subunits. Consistent with the proposed orientation of FapH, the N-terminus is closely associated with the OM, which would allow the acyl side chains of the FapH lipoprotein to insert into the OM.

### Identification of FapH interaction partners

**Fig 5:**
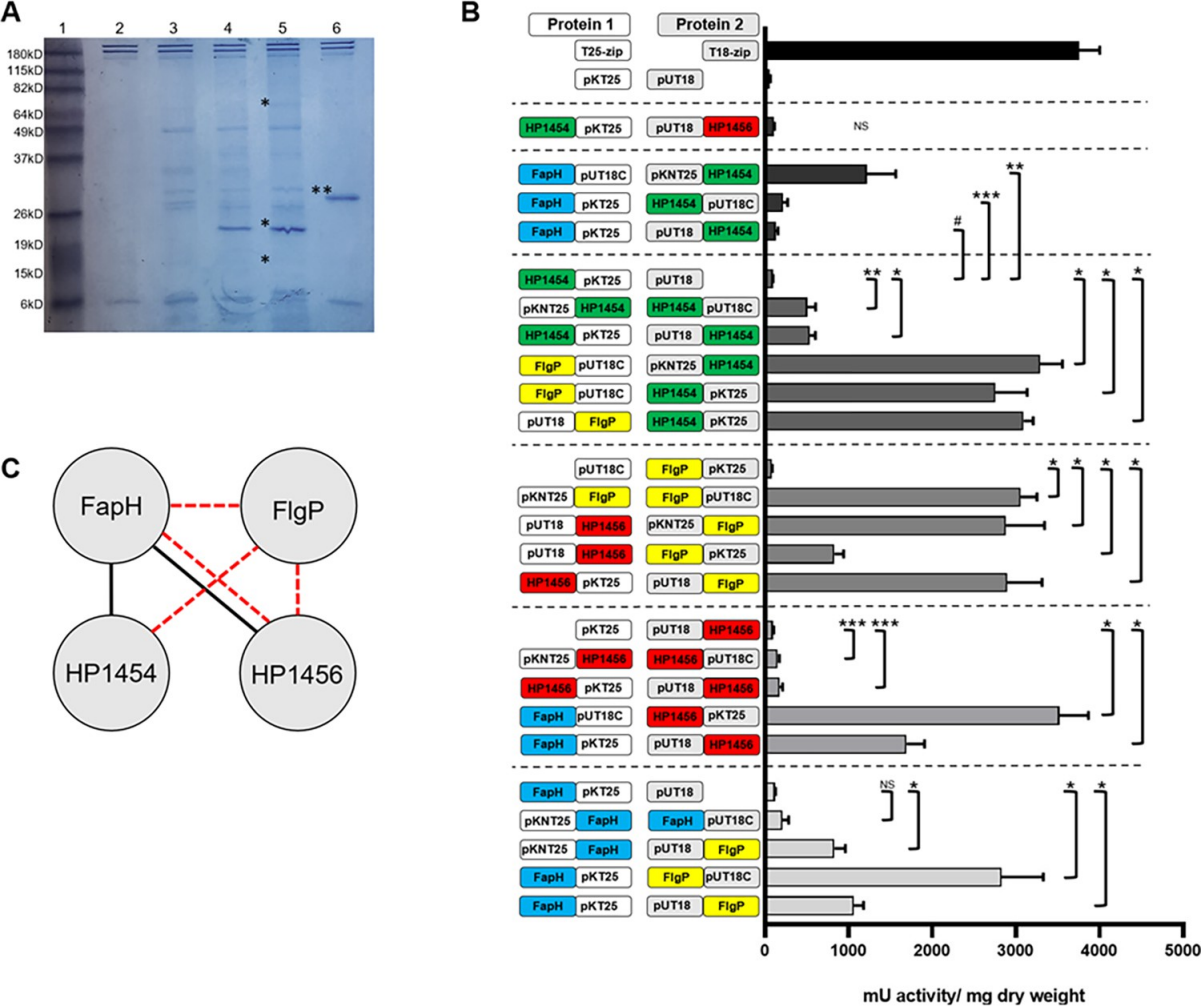
Identification of FapH interaction partners. (**A**) SDS-PAGE gel of proteins isolated from co-IP assay using anti-myc antibody. Lane 1 contained the molecular weight standards. Sizes of the protein standards are indicated to the left of the gel. Lanes 2 and 3 are negative controls and are samples from the co-IP assay with cellular extracts prepared from wild type *H*. *pylori* B128 and correspond to 5 μl and 10 μl of loaded sample, respectively. Lanes 3 and 4 are samples from the co-IP assay with cellular extracts prepared *H*. *pylori* B128 strain expressing the FapH-myc fusion protein and correspond to 5 μl and 10 μl loaded sample, respectively. Lane 6 shows a c-myc tagged *E*. *coli* protein (indicated by **) that served as a positive control for the co-IP assay and was supplied with the Pierce c-Myc Tag Magnetic IP/co-IP kit. Protein bands in lane 5 that were excised and analyzed to identify proteins present are indicated by an asterisk (*). HopD and FtsH were identified in the top band, the middle band corresponded to the FapH-myc protein, and HP1456 was identified in the lower band. (**B**) Results from β-galactosidase assays for strains with select combinations of adenylate cyclase T15 and T25 fragments fused to FapH, FlgP, HP1454, and HP1456. The cartoons shown on the y-axis illustrate the orientation of the adenylate cyclase fragments and *H*. *pylori* proteins in the fusion proteins relative to each other. In the cartoons, FapH is indicated in blue, FlgP is indicated in yellow, HP1456 is indicated in red, and HP1454 is indicated in green. pKNT25 and pKT25 indicate the adenylate cyclase T25 fragment at the N-terminus or C-terminus of the fusion protein, respectively. pUT18 and pUT18C indicate the adenylate cyclase T18 fragment at the N-terminus or C-terminus of the fusion protein, respectively. An *E*. *coli* strain containing the BACTH vectors pKT25 and pUT18 served as a negative control, and an *E*. *coli* strain bearing plasmids that expressed the T25-zip andT18-zip proteins served as a positive control. The bars indicate the average β-galactosidase activity for each strain and the error bars indicate the SEM. At least 3 replicates were done for each sample. A single asterisk (*) indicates a *p*-value of < 0.0001, two asterisks (**) indicate a *p*-value of < 0.001, and three asterisks (***) indicated a *p*-value of < 0.005 as determined using a two-sample *t* test. Significance of interaction between pKNT25-FlgP and FlgP-pUT18C is compared with pKT25 and pUT18. For the FapH-FlgP interactions, 7 of the 8 possible plasmid combinations produced significantly elevated levels of β-galactosidase activity, but only 3 of the 7 strains are shown in the figure. (**C**) Protein-protein interaction map constructed from co-IP and BACTH experiments. Solid black lines indicate interactions observed in co-IP assays involving the FapH-myc protein and dashed red lines indicate interactions observed in the BACTH assay.

We sought to identify proteins that interact with FapH to gain a better understanding of how FapH protects the barrier function of the OM from flagellum-mediated damage. Moreover, given the close association of the FapH ring and outer disk, we reasoned that identified FapH interaction partners would be good candidates for outer disk components. FapH was reported to interact with HP1409/HP0426 (these proteins are duplicated in *H*. *pylori* 26695) and the basal disk protein FlgP in a high-throughput yeast two-hybrid screen [30]. HP1409 and HP0426 are predicted cytoplasmic proteins and are unlikely to interact with FapH within the context of the flagellar basal body. To identify additional FapH interaction partners, we expressed a c-Myc-tagged FapH protein in ΔfapH pflA* and performed co-immunoprecipitation (co-IP) assays with cell extracts of the strain. Examination of the immunoprecipitated proteins by SDS-PAGE followed by Coomassie blue staining revealed a few prominent protein bands in the sample that contained the FapH-Myc fusion protein (**Fig 5A**, lanes 4 and 5) that were not apparent in the sample prepared with cell extracts from wild type *H*. *pylori* B128, which served as a negative control (**Fig 5A**, lanes 2 and 3). Proteins in three of these bands were identified by peptide mass fingerprinting. FapH was the only identified protein in the ~26-kDa band, which was the most intense band on the gel. Two proteins, HopD and FtsH, were identified in the ~70-kDa band. HopD is one of five β-barrel OM proteins (HopA, HopB, HopC, HopD, and HopE) in *H*. *pylori* that are identified as porins and are structurally homologous with the *E*. *coli* OmpF porin [31]. FtsH is an integral membrane, ATP-dependent zinc metallopeptidase that functions in quality control of integral membrane proteins [32]. HP1456 (also referred to as Lpp20), a predicted lipoprotein of unknown function, was the only identified protein in the ~15-kDa band. A crystal structure for *H*. *pylori* HP1456 indicates that the protein is elongated and bent, consisting of a three-stranded anti-parallel β-sheet flanked by three α-helices on one side and a short α-helix on the other side [33]. Drobnič and co-workers reported that the predicted tertiary structures of *C*. *jejuni* FlgP and *Vibrio alginolyticus* FlgT are very similar to that of *H*. *pylori* HP1456 [27]. Like FlgP, FlgT forms a disk-like motor accessory [34].

Additional proteins that were pulled-down in the co-IP assays used for the fingerprinting analysis, as well as co-IP samples from two additional replicates were identified by analyzing the total protein content of the samples by mass spectroscopy (**S3 Table**). Notably, HP1456 was identified in all three replicates of the co-IP assay and FlgP was identified in one of the three replicates. HP1454, which is encoded in the same operon as HP1456, was identified in two of the replicates of the co-IP assay. HP1454 is a secreted protein composed of three distinct domains [35], the first of which is structurally similar to HP1456 [33].

Since FlgP was identified in one of the replicates of the co-IP assay with the FapH-Myc fusion protein, we used a co-IP assay to determine if FapH was pulled-down with FlgP and to also identify other potential FlgP partners. For these co-IP assays, we used cell extracts prepared from a *H*. *pylori* B128 Δ*flgP* mutant that expressed FlgP-myc fusion protein. A caveat of the experiment was the Δ*flgP* mutant was aflagellated. WGS of the Δ*flgP* mutant revealed that the strain had a single nucleotide deletion within a homopolymeric tract of eight guanosines in *fliP*, which encodes a component of the flagellar protein export apparatus. Slipped-strand mispairing-mediated mutagenesis within this poly(G)-tract in *fliP* was reported previously for *H*. *pylori* 26695, and was suggested as a mechanism for switching between “on” and “off” phases for flagellation and motility [36]. Despite the mutation in *fliP*, we proceeded with co-IP assays with the expectation that FlgP can associate with at least some of its interaction partners in the absence of the axial components of the motor. Mass spectrometry analysis of the proteins pulled-down in two biological replicates of the co-IP assay with the FlgP-myc protein identified several potential FlgP interaction partners (**S3 Table**). Notably, FapH was identified in both replicates of the co-IP assay. Other proteins of interest identified from the co-IP assay with the FlgP-myc protein included HP1456 and HP1454.

To examine the validity of the protein interactions predicted from the co-IP assays, we examined the interactions of FapH with selected proteins using the bacterial adenylate cyclase two-hybrid (BACTH) assay [37]. The BACTH assay is based on the reconstitution of adenylate cyclase (CyaA) activity where two complementary fragments from the catalytic domain of the *Bordetella pertussis* CyaA toxin are brought into proximity by interacting proteins that are fused to the CyaA fragments. Reconstitution of CyaA activity leads to production of cAMP, which is assessed by monitoring the expression of cAMP-dependent genes in an *E*. *coli cyaA* mutant. We constructed vectors that expressed FapH, HP1454, HP1456, or FlgP fused to the N- or C-termini of the *B*. *pertussis* CyaA fragments T18 and T25. In constructing the expression vectors, the sequences encoding the signal peptides of the *H*. *pylori* proteins were omitted to avoid secretion of the fusion proteins into the periplasm.

All possible combinations of expression vectors in the *E*. *coli* reporter strain BTH101 were examined by culturing the cells on McConkey agar medium that contained 1% maltose (**S6 Fig**), and β-galactosidase activities were measured for strains with plasmid combinations that produced pink or red colonies on McConkey-maltose agar medium (**Fig 5B**). Consistent with the co-IP data that suggested interactions between FapH and HP1456, five of the eight combinations of FapH-CyaA and HP1456-CyaA fusion proteins reconstituted active CyaA as demonstrated by the significantly higher β-galactosidase activities in strains harboring these vector combinations versus the negative control (**Fig 5B**). In addition, three of the eight combinations of FapH-CyaA and HP1454-CyaA fusion proteins reconstituted active CyaA (**Fig 5B**). Consistent with the results from the co-IP assays with both the FapH-myc and FlgP-myc proteins as well as the reported interactions between FlgP and FapH in a yeast two-hybrid assay [30], seven of the eight combinations of FapH-CyaA and FlgP-CyaA fusion proteins reconstituted active CyaA (**Fig 5B**). FlgP also interacted with HP1454 and HP1456 in the BACTH system, as well as itself (**Fig 5B**). The strong interaction of FlgP with itself is consistent with the assembly of FlgP monomers to form the basal disk. Additional results from the BACTH assays suggest that HP1454 interacts weakly to moderately with itself but does not interact with HP1456 (**Fig 5B**). In addition to interacting with FlgP and FapH, HP1456 interacted weakly with itself (**Fig 5B**). Results of the protein-protein interaction studies are summarized in **Fig 5C**.

Given the interactions between FapH and HP1456 in the co-IP and BACTH assays (**Fig 5**) as well as the structural similarity between HP1456 and the disk-forming motor accessory proteins FlgP and FlgT [27], HP1456 seemed a good candidate for an outer disk protein. To address the hypothesis that HP1456 forms the outer disk of the *H*. *pylori* motor, we generated an unmarked deletion of *hp1456* in *H*. *pylori* B128 and characterized the resulting mutant. The number of flagella produced by the Δ*hp1456* mutant and the motility of the mutant in soft agar medium were the same as observed for wild type (**S7A-S7C Fig**). In addition, the Δ*hp1456* mutant displayed wild-type resistance to bacitracin (**S7D Fig**), indicating that HP1456 is not required by FapH to protect the barrier function of the OM from flagellum-mediated damage. The *in-situ* structure of the Δ*hp1456* mutant motor was indistinguishable from that of wild type (**S7E and S7F Fig**), indicating that HP1456 is not required for assembly of the outer disk. Thus, despite being a promising candidate, HP1456 is not an outer disk component.

## Discussion

The flagellar motors of *H*. *pylori* and other bacteria contain accessory structures that are not present in the archetypal *E*. *coli* and *S*. *enterica* motors [10,13–15]. The functions and protein components for many of these motor accessories have yet to be determined. We report here on a previously uncharacterized ring-like structure in the *H*. *pylori* motor that is associated with the OM and is dependent on the lipoprotein FapH for its assembly. In the absence of the FapH ring, *H*. *pylori* displays an increased sensitivity to bacitracin that is dependent on a functional flagellum. In support of this thesis, the motile ΔfapH pflA* strain, but not the non-motile, parental strain Δ*fapH pflA*^T^, displayed increased sensitivity to bacitracin compared to wild type (**Fig 3B–3D**). Moreover, introducing *fapH* into the ΔfapH pflA* strain on a shuttle vector suppressed the antibiotic sensitivity of the strain (**Fig 3B–3D**), indicating that loss of *fapH* was responsible for the increased antibiotic sensitivity of the strain. Although the PflA* variant has a 12 amino acid sequence that differs from the wild-type sequence, a couple of lines of evidence indicate this alteration in PflA is neither responsible nor required for the antibiotic sensitivity of the ΔfapH pflA* mutant. Firstly, introducing a plasmid-borne copy of *fapH* into ΔfapH pflA* suppressed the bacitracin-sensitivity of the strain, indicating the PflA* variant is not responsible for the antibiotic sensitivity of the strain. Secondly, the Δ*fapH* mutants that were generated subsequent to the isolation and characterization of ΔfapH pflA* were sensitive to bacitracin and have the wild-type *pflA* allele, indicating that the PflA* variant is not required for the antibiotic sensitivity of the *H*. *pylori fapH* mutant.

Bacitracin inhibits peptidoglycan biosynthesis by binding undecaprenyl-pyrophosphate within the periplasmic space [29] and is normally excluded by the OM. In various bacterial species, mutations that lead to the accumulation of GPLs in the outer leaflet of the OM result in increased sensitivity to bacitracin and other antibiotics due to the increased permeability of the OM to these compounds [17,38,39]. The increased sensitivity of ΔfapH pflA* and Δ*fapH* mutants to bacitracin suggests the lipid asymmetry of the OM is compromised in these strains. We hypothesize that in the absence of the FapH ring, stress exerted on the OM and flagellar sheath by the rotation of the flagella and the counterrotation of the cell body facilitates the intrusion of GPLs into the outer leaflet of the OM and compromises the barrier function of the OM.

Given the importance of lipid asymmetry for OM barrier function, bacteria have evolved various systems for removing GPLs that make their way into the outer leaflet of the OM. One mechanism for removing GPLs from the outer leaflet of the OM in *E*. *coli* and other bacteria is the maintenance of lipid asymmetry (Mla) system, which consists of the OM lipoprotein MlaA, the periplasmic chaperone MlaC, and the inner membrane complex MlaFEDB [40]. MlaA extracts GPLs from the outer leaflet of the OM and transfer them to MlaC for transport across the periplasmic space to the MlaFEDB complex, which inserts the GPLs into the inner membrane [40]. *H*. *pylori* possesses homologs of MlaF (HP1465), MlaE (HP1466), and MlaD (HP1464) but lacks a MlaB homolog as has been reported for other members of the phylum Campylobacterota [39]. Although homologs of MlaA and MlaC have been reported in *C*. *jejuni* 11168 and 488 [38], *H*. *pylori* lacks homologs to both of these proteins, suggesting that the *H*. *pylori* Mla system differs somewhat from the well-characterized Mla system in *E*. *coli* and other bacteria. A second mechanism used by *E*. *coli* to remove GPLs from the outer leaflet of the OM is the enzyme PagP, which acylates the lipid A moiety of LPS at the position 2 R-3-hydroxymyristate chain with a GPL-derived palmitoyl group [41]. *H*. *pylori*, however, lacks a PagP homolog.

A third mechanism for removing GPLs from the outer leaflet of the OM is the phospholipase PldA, which catalyzes the hydrolysis of acyl ester bonds in GPLs that enter the outer leaflet of the OM to generate lysophospholipids and free fatty acids [42–44]. *H*. *pylori* possesses a PldA homolog, but aspects of the enzyme differ somewhat from *E*. *coli* PldA. For example, *H*. *pylori* PldA is proposed to be a virulence factor as a *H*. *pylori* SS1 *pldA* mutant was unable to colonize mice [45]. *H*. *pylori* PldA also appears to have additional enzymatic activities as the *H*. *pylori* SS1 *pldA* mutant was reported to have reduced phospholipase activity, as well as reduced lecithinase and hemolytic activities compared to the parental strain [45]. The lecithinase activity was assessed by observing zones of clearing around *H*. *pylori* grown on egg yolk agar [45], suggesting PldA was secreted in amounts that were sufficient to hydrolyze the egg yolk lecithin. Expression of *H*. *pylori pldA* is phase variable as a result of slippage during DNA replication within a homopolymeric (G)-tract located about two-thirds of the way into the open reading frame of the gene [46]. Changes in the length of the (G)-tract result in reversible frameshifts, which manifest in translation of the full-length, active PldA or a truncated, inactive enzyme [46].

The *H*. *pylori* B128 genome sequence in the NCBI database indicates the poly(G)-tract in *pldA* is 7 nucleotides in length, whereas the poly(G)-tract in the wild-type B128 strain used in our studies is 10 nucleotides in length. In both cases though, *pldA* is predicted to be in the ‘off’ phase. WGS of the Δ*fapH pflA*^T^ and ΔfapH pflA* strains indicated the poly(G)-tract in *pldA* increased from 10 to 11 nucleotides in 67% and 63% of the reads for that region, respectively (**S2 Table**), indicating that *pldA* had switched to the ‘on’ phase in most of the cells in the populations of these strains. Increased PldA activity resulting from *pldA* switching to the ‘on’ phase may have compensated for any breakdown in the OM lipid asymmetry. WGS of the Δ*fapH* mutant and its parental *fapH*::kan^R^-*sacB* strain, however, revealed that while the poly(G)-tract in *pldA* was reduced from 10 to 9 nucleotides in >90% of the reads for that region (**S2 Table**), *pldA* remained in the ‘off’ phase in these strains. It is possible that reducing the poly(G)-tract to 9 nucleotides allowed for low level expression of full-length PldA due to the ribosome shifting one nucleotide backward (-1, in the direction of the 5’-end of the transcript) while translating the poly(G)-tract. Translational frameshifting results from the ribosome shifting either one nucleotide forward (+1, in the direction of the 3’-end of the transcript) or one nucleotide backward, but most case studies on translational frameshifting involve -1 shifting [47]. Although increased PldA activity in the *fapH* mutants may have helped to mitigate disruptions in OM lipid asymmetry, the Mla pathway plays a more critical role in maintaining the lipid asymmetry of the OM in *E*. *coli* as loss the Mla pathway results in a slight increase in OM permeability, whereas loss of PldA has no effect [40]. The apparent disruption in OM barrier function in the ΔfapH plfA* and Δ*fapH* mutants suggest the systems for maintaining OM lipid asymmetry were overwhelmed in these strains. Alternatively, the disruption in lipid asymmetry of the OM in the *fapH* mutants may have been localized near the flagella and OM lipid asymmetry was maintained over the bulk of the cell surface.

An intriguing question is what is the mechanism by which the FapH ring protects the OM from flagellum-dependent damage? One possibility is that the lipid moieties of FapH secure GPLs in the inner leaflet of the OM and prevent them from flipping into the outer leaflet. Additional *H*. *pylori* motor accessories associated with the OM, including the basal disk and outer disk, may function similarly to protect the OM from flagellum-mediated damage. The *C*. *jejuni* and *H*. *pylori* basal disk protein FlgP is a predicted lipoprotein. A high-resolution *in-situ* structure of the *C*. *jejuni* flagellar motor revealed that the basal disk is comprised of a dozen or more concentric rings, with the innermost ring consisting of 17 trimeric repeats of 51 FlgP protomers [27]. Thus, the *H*. *pylori* basal disk is predicted to be formed by hundreds of FlgP protomers, and such an extensive network of lipoproteins may stabilize the OM around the base of the flagellum.

An alternative mechanism by which the FapH ring may protect the barrier function of the OM from flagellum-mediated damage is that it acts as a bushing and/or bearing (i.e., analogous function to the L-ring) to balance the flagellum as it rotates. The FapH ring is positioned near the base of the hook and may keep the hook aligned as the flagellum rotates to help to protect the integrity of the OM and flagellar sheath in this region.

Although FlgP, HP1456, and HP1454 were identified as FapH interaction partners in the co-IP assays and BACTH assays (**Fig 5**), the significance of these protein-protein interactions is unclear. It is possible that FapH recruits HP1456 and/or HP1454 to the base of the sheath to assist in stabilizing the region as the flagella rotate. If that is the case, the recruitment of HP1456 does not appear to be a major contributing factor to OM stability since the Δ*hp1456* mutant displayed wild-type resistance to bacitracin (**S7 Fig**). FlgP has a large unstructured region at the N-terminus that allows the lipoprotein to anchor in the OM and form the basal disk well below the OM [27] (also see **Fig 4A–4C**). Thus, although the basal disk is not intimately associated with the FapH, the unstructured N-terminal region for some of the FlgP protomers are positioned to associate potentially with the FapH ring. Moreover, little is known about the assembly of the flagellar motor accessories in *H*. *pylori*, and interactions between FapH and FlgP may be important for FapH ring assembly.

While the flagellar sheath may potentiate flagellum-mediated damage to the OM in *H*. *pylori*, flagellar rotation may also present problems in maintaining the barrier function of the OM in diderms that have sheath-less flagella. Since FapH is restricted to members of the phylum Campylobacterota, bacterial species outside the phylum presumably rely on different mechanisms to protect the OM from flagellum-mediated damage. In bacteria that lack a flagellar sheath, the L-ring forms a pore in the OM through which the flagellar rod passes, which may afford protection to the OM as the flagellum rotates. A high-resolution structure of the *S*. *enterica* flagellar basal body revealed that the LPS acyl chains and lipid moieties of FlgH form a hydrophobic band that encompasses the L-ring and is thinner than the canonical lipid bilayer [18]. An additional protein density was associated with the outside of the *S*. *enterica* L-ring, and this protein was identified as the lipoprotein YecR [18]. The authors of the study postulated a potential role for YecR in L-ring assembly, but their analysis of a *yecR* mutant indicated that YecR was not required for formation of the L-ring [18]. It is possible that like FapH, YecR has a role in protecting the OM from flagellum-mediated damage.

As our knowledge of the structural diversity of bacterial flagellar motors has grown, it has become clear that more work needs to be done to understand the roles of flagellar motor accessory structures. The characterization of motor accessories will undoubtedly lead to the discovery of novel mechanisms for regulating motor function or mitigating cellular damage resulting from action of the motor as we demonstrated here for the FapH ring of the *H*. *pylori* motor. Such new information will provide insight into how bacteria have sculpted the motor to address the demands imposed on them by specific ecological niches or by morphological variations in flagellum structure, such as the flagellar sheath.

## Materials and methods

### Bacterial strains and culture conditions

*E*. *coli* Turbo cells (New England Biolabs, Ipswich, MA, USA, which were used for cloning procedures, were grown on LB medium supplemented with ampicillin (100 μg/ml) or kanamycin (30 μg/ml) as required. *H*. *pylori* strains used in the study were derived from *H*. *pylori* B128 (kindly provided by Richard M. Peek, Jr.). and are listed along with plasmids used in the study in **S4 Table**. Liquid cultures of *H*. *pylori* were grown at 37°C with shaking in brain heart infusion (BHI) supplemented with 5% heat-inactivated horse serum (Gibco; Thermo Fisher Scientific, Waltham, MA, USA) in serum bottles that contained an atmosphere consisting of 5% CO_2_, 10% H_2_, 10% O_2_, and 75% N_2_. *H*. *pylori* strains were grown on solid medium under an atmosphere consisting of 10% CO_2_, 6% O_2_, and 84% N_2_ at 37°C on tryptic soy agar supplemented with 5% heat-inactivated horse serum (TSA-HS). Growth medium for *H*. *pylori* was supplemented with kanamycin (30 μg/ml), bacitracin (100 or 200 μg/ml), or 5% sucrose (w/v) as needed.

### PCR methods

Genomic DNA (gDNA) from *H*. *pylori* B128 was purified using the Wizard genomic DNA purification kit (Promega, Madison, WI, USA) and used as the template for PCR using PrimeSTAR DNA polymerase (Takara Bio, San Jose, CA, USA) or Phusion DNA polymerase (New England Biolabs). To facilitate cloning into the pGEM-T Easy vector (Promega), amplicons were incubated with *Taq* polymerase (Promega) at 72° C for 10 min to add A overhangs at the 3’-ends.

### Construction of *H*. *pylori* B128 Δ*fapH*, Δ*flgP*, and Δ*hp1456* mutants

A schematic illustrating the process for deleting *fapH* in the *H*. *pylori* B128 chromosome is shown in **S8 Fig**, and the same general approach was used to delete *flgP* and *hp1456*. Regions flanking *fapH* were amplified from *H*. *pylori* B128 gDNA using primer pair 59 and 60 (for upstream region) and primer pair 61 and 62 (for downstream region; **S5 Table**). Primers 60 and 61 were complementary at their 5’-ends and introduced XhoI and NheI restriction sites for subsequent introduction of a kan^R^-*sacB* cassette. The amplicons corresponding to the regions upstream and downstream of *fapH* were joined together by overlapping PCR, A overhangs were added to the 3’-ends of the resulting amplicon, which was then ligated into pGEM-T Easy (Promega) to generate plasmid pKR10. Plasmid pKR3 is a derivative of pJC038 [48] that carries a kan^R^*-sacB* cassette where *sacB* is under control of the *H*. *pylori ureA* promoter. In pKR3, the *H*. *pylori ureI* promoter was introduced at the end of the cassette in an outward orientation to compensate for potential polar effects on genes that are downstream of the kan^R^-*sacB* insertion. The kan^R^-*sacB* cassette was excised from plasmid pKR3 by digesting with NheI and XhoI (New England Biolabs) and then introduced into the NheI and XhoI sites in plasmid pKR10 to generate plasmid pKR11, which was introduced by natural transformation into *H*. *pylori* B128. Since plasmid pKR11 does not replicate in *H*. *pylori*, transformants in which *fapH* had been replaced with the kan^R^-*sacB* cassette by homologous recombination were enriched by selecting for kanamycin resistance. Replacement of *fapH* with the kan^R^-*sacB* cassette was confirmed by PCR using primer pair 59 and 62. One of the kanamycin-resistant isolates in which *fapH* was replaced with the kan^R^-*sacB* cassette, which was designated strain H6, was transformed with the suicide vector pKR10. Transformants in which the kan^R^-*sacB* cassette was replaced with the unmarked deletion of *fapH* resulting from homologous recombination between plasmid pKR10 and the chromosome were counter-selected on TSA-HS supplemented with 5% sucrose as described [49]. Sucrose-resistant isolates were screened for kanamycin-sensitivity on TSA-HS supplemented with kanamycin, and deletion of *fapH* in kanamycin sensitive isolates was confirmed by PCR using primer pair 59 and 62 and DNA sequencing of the resulting amplicon (Eton Biosciences, Research Triangle, NC, USA). A *H*. *pylori* B128 strain in which *fapH* was deleted was designated as strain H16.

An unmarked deletion of *flgP* in *H*. *pylori* B128 was constructed following the protocol used for constructing the Δ*fapH* mutant. Regions flanking *flgP* were amplified from *H*. *pylori* B128 gDNA using primer pair 39 and 40, and primer pair 41 and 42. The resulting amplicons were joined together by overlapping PCR and cloned into pGEM-T Easy to generate plasmid pKR4. The kan^R^-*sacB* cassette was introduced into plasmid pKR4 to generate the suicide vector pKR9, which was introduced into *H*. *pylori* B128 to replace *flgP* with the kan^R^-*sacB* cassette through homologous recombination to yield strain H5. Replacement of *flgP* with the kan^R^-*sacB* cassette was confirmed by PCR using primer pair 39 and 42. The suicide vector pKR4 was introduced into H5 to replace the kan^R^-*sacB* cassette in the *flgP* locus with the unmarked deletion of *flgP* in plasmid pKR4 using the sucrose counter-selection as described above. Sucrose-resistant isolates were screened for kanamycin-sensitivity on TSA-HS supplemented with kanamycin, and deletion of *flgP* in kanamycin sensitive isolates was confirmed by PCR using primer pair 39 and 42 and DNA sequencing of the resulting amplicon. A *H*. *pylori* B128 strain in which *flgP* was deleted was designated as strain H6. gDNA from strain H6 was prepared and provided to the SeqCenter (Pittsburgh, PA, USA) for whole genome Illumina sequencing. The resulting genome sequence data were analyzed using the *breseq* computational pipeline [50].

An unmarked deletion of *hp1456* in *H*. *pylori* B128 was constructed following the protocol used for constructing the Δ*fapH* mutant. Regions flanking *hp1456* were amplified from *H*. *pylori* B128 gDNA using primer pair 195 and 196, and primer pair 197 and 198. The resulting amplicons were joined together by overlapping PCR and cloned into pGEM-T Easy to generate plasmid pKR67. The kan^R^-*sacB* cassette was introduced into plasmid pKR67 to generate the suicide vector pKR77, which was introduced into *H*. *pylori* B128 to replace *hp1456* with the kan^R^-*sacB* cassette through homologous recombination to yield strain H143. Replacement of *hp1456* with the kan^R^-*sacB* cassette was confirmed by PCR using primer pair 195 and 198. The suicide vector pKR67 was introduced into H143 to replace the kan^R^-*sacB* cassette in the *hp1456* locus with the unmarked deletion of *hp1456* in plasmid pKR67 using the sucrose counter-selection as described above. Sucrose-resistant isolates were screened for kanamycin-sensitivity on TSA-HS supplemented with kanamycin, and deletion of *hp1456* in kanamycin sensitive isolates was confirmed by PCR using primer pair 195 and 198 and DNA sequencing of the resulting amplicon. A *H*. *pylori* B128 strain in which *hp1456* was deleted was designated as strain H145. gDNA from strain H145 was prepared and provided to the SeqCenter (Pittsburgh, PA, USA) for whole genome Illumina sequencing. The resulting genome sequence data were analyzed using the *breseq* computational pipeline [50].

An unmarked deletion of *pflA* in *H*. *pylori* B128 was constructed following the protocol used for constructing the Δ*fapH* mutant. Regions flanking *pflA* were amplified from *H*. *pylori* B128 gDNA using primer pair 43 and 44, and primer pair 45 and 46. The resulting amplicons were joined together by overlapping PCR and cloned into pGEM-T Easy to generate plasmid pKR5. The kan^R^-*sacB* cassette was introduced into plasmid pKR5 to generate the suicide vector pKR7, which was introduced into *H*. *pylori* B128 to replace *pflA* with the kan^R^-*sacB* cassette through homologous recombination to yield strain H3. Replacement of *pflA* with the kan^R^-*sacB* cassette was confirmed by PCR using primer pair 43 and 46. The suicide vector pKR7 was introduced into H3 to replace the kan^R^-*sacB* cassette in the *pflA* locus with the unmarked deletion of *pflA* in plasmid pKR5 using the sucrose counter-selection as described above. Sucrose-resistant isolates were screened for kanamycin-sensitivity on TSA-HS supplemented with kanamycin, and deletion of *pflA* in kanamycin sensitive isolates was confirmed by PCR using primer pair 43 and 46 and DNA sequencing of the resulting amplicon. A *H*. *pylori* B128 strain in which *pflA* was deleted was designated as strain H7. gDNA from strain H7 was prepared and provided to the SeqCenter (Pittsburgh, PA, USA) for whole genome Illumina sequencing and the resulting genome sequence data were analyzed using the *breseq* computational pipeline.

### Complementation of mutants

To facilitate complementation assays in *H*. *pylori*, we modified the shuttle vector pHel3 [51] for Golden Gate cloning that employed tandem sites for the Type IIS restriction enzyme BspQ1 (**S9A Fig**). The modified plasmid, which we designated pHel3-GG, contains the *fliF* promoter upstream of the tandem BspQ1 sites where the cloned gene is introduced. The *fliF* promoter is flanked by unique BamHI and NheI sites that can be used to replace the *fliF* promoter with other promoters if desired.

Primers 169 and 170 were used to amplify *fapH* from *H*. *pylori* B128 gDNA (**S5 Table**). The primers introduced BspQ1 sites immediately upstream and downstream of the start and stop codons of *fapH*, respectively. The resulting amplicon and pHel3-GG were digested together with BspQ1 (New England Biolabs) for 1 h at 50°C, after which the amplicon and vector were ligated using Fast-Link DNA ligase (Biosearch Technologies, Hoddeson, UK) for 30 min at room temperature. The reaction mix was incubated with BspQ1 to digest any uncut pHel3-GG vector, and the reaction mix was then used for transformation of *E*. *coli*. A plasmid containing the expected insert, designated as pKR54, was verified by restriction enzyme digestion and sequencing of the inserted DNA.

Primers 159 and 160 were used to amplify *pflA* from *H*. *pylori* B128 gDNA. The primers introduced BspQ1 sites immediately upstream and downstream of the start and stop codons of *pflA*, respectively. The resulting amplicon was cloned into pHel3-GG as described above. A plasmid containing the expected insert, designated as pKR50, was verified by restriction enzyme digestion and sequencing of the inserted DNA. Plasmids pKR54 and pKR50 were introduced by natural transformation into various *H*. *pylori* strains for complementation assays.

### Construction of c-Myc tagged FapH and FlgP proteins

To facilitate construction of a c-Myc-tagged FapH in *H*. *pylori*, we modified the shuttle vector pHel3 [51] to allow for the introduction of the c-Myc epitope at the C-terminus of the protein (**S9B Fig**). The modified plasmid, which we designated pHel3-Myc, contains the *fliF* promoter upstream of the tandem BspQ1 sites for Golden Gate cloning followed by a sequence encoding a flexible linker, c-Myc epitope, and DDDDK epitope. Primers 162 and 163 were used to amplify *fapH* from *H*. *pylori* B128 gDNA (**S5 Table**). The primers introduced BspQ1 sites immediately upstream and downstream of the start and stop codons of *fapH*, respectively. The resulting amplicon and plasmid pHel3-Myc were digested together with BspQ1 and ligated using Fast-Link DNA ligase as described above. The reaction mix was incubated with BspQ1 to digest uncut pHel3-Myc vector and the reaction mix was then used for transformation of *E*. *coli*. A plasmid containing the expected insert, designated as pKR55, was verified by restriction enzyme digestion and sequencing of the inserted DNA.

To construct a c-Myc-tagged FlgP, primers 78 and 79 were used to amplify *flgP* from *H*. *pylori* B128 gDNA. The resulting amplicon and plasmid pHel3-Myc were digested with BspQ1 and ligated together to generate plasmid pKR23. The *flgP*-*myc* chimeric gene in plasmid pKR23 was confirmed by DNA sequencing.

### Motility assay

Motility was evaluated using a semisolid medium containing Mueller-Hinton broth (MHB), 10% heat-inactivated horse serum, 20 mM MES (2-(4-morpholino)-ethane sulfonic acid) (pH 6.0), and 0.4%-0.6% Noble agar. A minimum of three technical replicates were used to assess the motility of each strain. *H*. *pylori* strains grown on TSA-HS for 2 d were stab-inoculated into the motility agar and incubated at 37° C under an atmospheric condition consisting of 10% CO_2_, 6% O_2_, and 84% N_2_. The diameters of the resulting swim halos were measured 7 d post-inoculation and a two-sample t-test was used to determine statistical significance. For motility-base antibiotic resistance assays, bacitracin was included in the soft agar medium at the indicated concentrations.

### Efficiency plating assay

*H*. *pylori* strains were grown on TSA-HS, then resuspended in MHB. The densities of each cell suspension were adjusted to an OD_600_ of ~0.50. Cell suspensions were diluted serially, 3 μl of each dilution were plated onto TSA-HS and TSA-HS supplemented with bacitracin, and the cultures were incubated for 5 d under a microaerobic atmosphere. A minimum of two replicants were done for each strain and tested condition.

### Co-immunoprecipitation procedure

Wild-type *H*. *pylori* B128 and the *H*. *pylori* strains expressing either the FapH-Myc or FlgP-Myc fusion proteins were grown on TSA-HS plates, harvested, and resuspended in 7 ml phosphate-buffer saline (PBS) with Pierce Protease Inhibitor (Thermo Fisher Scientific). Cells were lysed at 18,000 psi with a French press and the resulting cell lysate was centrifuged at 7,700 x g for 10 min to remove unlysed cells and cell debris. The resulting supernatant was centrifuged at 100,000 x g to pellet membrane vesicles, which were subsequently resuspended in PBS buffer containing 50 mM n-dodecyl-β-D-maltoside (DDM) (Chem-Impex International, Wood Dale, IL, USA). The solution was diluted to 20 mM DDM and the insoluble material was pelleted by centrifugation at 10,000 x g for 10 min and the resulting supernatant was used for the co-IP procedure using the Pierce c-Myc Tag Magnetic IP/Co-IP kit (Thermo Fisher Scientific) as described by the supplier. Samples were incubated with the magnetic beads either overnight at 4°C or at room temperature for 30 min. Magnetic beads with bound proteins were washed at least 10 times with a 1:20 solution of Buffer 2 (supplied with the kit) containing 20 mM DDM. Proteins were eluted from the magnetic beads using 100 μl of 1x non-reducing sample buffer (supplied with the kit) followed by incubation at 95–100°C for 5 to 10 min. Samples were loaded on a 12% SDS-PAGE gel and proteins were visualized by staining with Coomassie Brilliant Blue R-250. Protein bands were excised from the gel and submitted to the University of Georgia Proteomics and Mass Spectrometry Facility for protein identification by peptide mass fingerprinting analysis. Proteomic analysis of total protein from the co-IP assays was done by loading the samples on a 12% SDS-PAGE gel and allowing the samples to enter the top of the resolving gel. The protein band was excised from the gel and submitted to UGA PAMS for analysis by in-gel trypsin digestion followed by LC-MS/MS on the Orbitrap mass spectrometer coupled with nano-HPLC using a 90-minute elution gradient.

### Transmission electron microscopy

*H*. *pylori* strains were grown to late-log phase (OD_600_ ~1.0) in MHB supplemented with 5% heat-inactivated horse serum. Cells from 1 mL of culture were pelleted by centrifugation (550 x g) then resuspended in 125 μL of PBS. Cells were fixed by adding 50 μL of 16% EM grade formaldehyde and 25 μL of 8% EM grade glutaraldehyde to the cell resuspension. Following incubation at room temperature for 5 min, 10 μL of the cell suspension were applied to a 300 mesh, formvar-coated copper grid and incubated at room temperature for 5 min. The cell suspension was wicked off the grids using a filter paper, and the grids were washed 3 times with 10 μL of water. Cells were stained by applying 10 μL of 1% uranyl acetate to the grids for 30 s. After removing the stain with filter paper, the grids were washed three times with 10 μL of water and then air-dried. Cells were visualized using a JEOL JEM 1011 transmission electron microscope. The number of flagella per cell were determined for at least 100 cells for each strain. Flagellar lengths were determined using Fiji ImageJ.

### Cryo-ET sample preparation

*H*. *pylori* strains were grown on Columbia agar plates supplemented with 5% horse red blood cells at 37°C under microaerobic conditions. Bacteria from the agar medium were resuspended in PBS and mixed with 10 nm of BSA gold tracers (Aurion, Wageningen, NL). The mixtures were deposited on freshly grow-discharged cryo-EM grids (Quantifoil R2/1a Cu 200 mesh) and then frozen into liquid ethane by using a manual plunger as described previously [15,48].

### Cryo-ET data collection and processing

Frozen-hydrated specimens were visualized in a 300kV Titan Krios G2 transmission electron microscope (Thermo Fisher Scientific) equipped with a K3 summit direct detection camera and a BioQuantum energy filter (Gatan). Tilt series images were acquired at 42,000x magnification (corresponding to a pixel size of 2.148 Å at the specimen level) and ~4.5 μm defocus using SerialEM [52] and FastTomo script [19] based on a dose-symmetric scheme from -48° to +48° in 3° increments [53]. The total accumulative dose for each tilt series was ~60 e^-^/Å^2^. MotionCor2 [54] was used to correct the beam induced motion. IMOD [55] was used to create image stacks and align all images in each tilt series by tracing the gold tracer beads. Gctf [56] was used for the defocus estimation for all images in the tilt series, and then the ctfphaseflip function in IMOD was used for contrast transfer function (CTF) correction [57].

### Subtomogram averaging

Tomo3D [58] was used to generate 3D reconstructions with the simultaneous iterative reconstruction technique (SIRT), and a total of 268, 225, and 146 tomograms were reconstructed from wild type, Δ*fapH pflA*^T^, and ΔfapH pflA*, respectively. 6 x binned tomograms were used to select 824, 640, and 534 flagellar motors from wild type, Δ*fapH pflA*^T^, and ΔfapH pflA*, respectively. After the particle picking, tomograms with weighted back projection (WBP) were used for the initial subtomogram averaging with the i3 suite [59,60]. For further structural analysis, 4 x binned subtomograms were used to refine the intact motor structures.

### FapH modeling in the intact flagellar motor

Alphafold 2 was used to generate a structural prediction for FapH [28,61]. The predicted structure deemed the best model was used to fit into the intact motor structure derived from subtomogram averaging by using the “fit in map” option in UCSF ChimeraX software [62]. All models examined had the signal peptides removed.

### Whole genome sequencing and analysis

gDNA from the *H*. *pylori* strains was purified using the Wizard genomic DNA purification kit (Promega) and submitted to the SeqCenter (Pittsburgh, PA, USA) for genomic library preparation and Illumina sequencing. Reads for *H*. *pylori* gDNA sequence were mapped using the *breseq* computational pipeline [50] with the published NCBI genome for *H*. *pylori* B128 (Accession no.: NZ_CP024951.1).

### Construction of plasmids for BACTH assays

The Bacterial Adenylate Cyclase Two-Hybrid (BACTH) System Kit (Euromedex, Souffelweyersheim, France) was used to detect and characterize protein-protein interactions *in vivo*. Primer pairs 208/209, 2010/211, 212/213, and 214/215 (**S5 Table**) were used to amplify *hp1454*, *hp1456*, *fapH*, and *flgP*, respectively, from *H*. *pylori* B128 gDNA. The forward primers corresponded to sequences immediately following the regions that encoded the predicted signal peptides to prevent secretion of the recombinant proteins. Following the addition of A’s to the 3’-ends, the resulting amplicons were ligated into pGEM-T Easy. The resulting plasmids were designated pKR69, pKR70, pKR71, and pRK72 (**S4 Table**), Tand the inserted DNA sequences were confirmed by sequencing. BamHI-KpnI fragments from these plasmids were cloned into BamHI and KpnI sites in the BACTH vectors pKT25, pKNT25, pUT18, andpUT18C) to generate plasmid pRK96 though pRK112. DNA fragments that were cloned into the BACTH vectors were sequenced to confirm that the plasmid constructs were correct. *E*. *coli* reporter strain BTH101 (Euromedex) was used for co-transformation of BACTH plasmids. Eight combinations of plasmids were co-transformed and screened in BTH101 for each of the following protein-protein interaction pairs: FapH-FlgP, FapH-HP1454, FapH-HP1456, FlgP-HP1454, FlgP-HP1456, HP1454-HP1456, FlgP-FlgP, HP1454-HP1454, and HP1456-HP1456, for a total of 72 different combinations. Transformants were plated onto McConkey agar supplemented with 1% (w/v) maltose, 100 μg/ml ampicillin, 50 μg/ml kanamycin, and 0.5 mM isopropyl-β-D-thiogalactopyranoside (IPTG).

### β-galactosidase assay

Cultures of *E*. *coli* BTH101 harboring various combinations of BACTH plasmids were grown at 30°C to mid- to late-log phase in LB supplemented with ampicillin (50 μg/ml), kanamycin (25 μg/ml), and 0.5 mM IPTG. Cells were diluted five-fold in M63 medium (15 mM (NH_4_)_2_SO_4_,100 mM KH_2_PO_4_, 1.8 μM FeSO_4_, 1 mM MgSO_4_, and 4 μM thiamine) containing 0.2% maltose as the carbon source in a final volume of 2.5 ml and OD_600_ readings were recorded for each culture. Cells were permeabilized by adding 30 μl of toluene and 35 μl of a 0.1% sodium dodecyl sulfate solution then incubating for 30–40 min at 37°C with shaking. The permeabilized cells were diluted 10-fold in PM2 buffer (70 mM Na_2_HPO_4_, 30 mM NaH_2_PO_4_, 1 mM MgSO_4_, 0.2 mM MnSO_4_, 100 mM β-mercaptoethanol, pH 7.0) to a final volume of 1 ml and placed in 5 ml glass tubes. Cells were incubated for 5 min in a water bath at 28°C, and the enzymatic reaction was started with the addition of 0.25 ml of 13 mM *o*-nitrophenol-β-galactoside (ONPG) in PM2 buffer. Enzyme reactions were stopped at various recorded times by the addition of 0.5 mL of 1 M Na_2_CO_3_, and then the OD_420_ values were recorded for each sample. The enzymatic activity (A; units (in nmol ONPG hydrolyzed min^-1^)/ml) was calculated according to the instructions provided by the BACTH kit supplier using the formula A = 200 x (OD_420_ / min of incubation) x dilution factor. The factor 200 in the formula is the inverse of the OD_420_ for 1 μM *o*-nitrophenol using a 1 cm light path, which is 0.0045 at pH 11.0. The dilution factor for each was 10. Specific activities (SA) expressed in units per mg dry weight were calculated by considering 1 ml of culture at OD_600_ = 1 corresponds to 300 μg dry weight bacteria and using the formula SA = A / (OD_600_ x 0.3 mg dry weight per OD_600_). Each strain was assayed with at least three replicates, and a two-sample *t*-test was used to determine statistical significance.

## Supporting information

S1 Fig Predicted tertiary structures of PflA, PflA^T^ and PflA*. Predicted structures generated by AlphaFold 2 [28] are shown for (**A**) native PflA, (**B**) truncated PflA expressed in Δ*fapH pflA*^*T*^, and (**C)** PflA* variant expressed in ΔfapH pflA*. Close ups of the regions where the PflA proteins differ are shown below each modeled structure. Red coloring indicates residues where mutations occurred and blue coloring indicates resides that are changed downstream of where the mutations occur.
(TIF)

S2 Fig Characterization of flagellation patterns and growth rates of Δ*fapH pflA*^T^ and ΔfapH pflA*. (**A**) Number of flagella per cell for *H*. *pylori* B128 wild type, Δ*fapH pflA*^T^, and ΔfapH pflA*. Cells were grown in liquid media, harvested, and visualized by TEM and the number of flagella per cell (n = 100) were counted for each strain. An ANOVA analysis of the data indicated there was no significant difference between the strains in the number of flagella per cell. ns–not significant. (**B**) Lengths of the flagellar filaments were measured using ImageJ and an ANOVA analysis of the data was done to assess the statistical significance of any differences. The asterisk (*) indicates a *p*-value < 0.005. (**C**) Growth rates of *H*. *pylori* strains in brain heart infusion (BHI) supplemented with 5% heat-inactivated horse serum were determined by measuring OD_600_ values of the cultures at various times. Calculated doubling times for the strains were: wild type B128–7.1 h; Δ*fapH pflA*^T^*—*6.6 h; ΔfapH pflA* - 13.1 h; ΔfapH pflA*/ p*fapH*—7.0 h. An ANOVA analysis of the data was done to assess the statistical significance of any differences. The double asterisk (**) indicates a *p*-value < 0.05.
(TIF)

S3 Fig Reconstructed Δ*fapH* mutants display sensitivity to bacitracin. (**A**) *H*. *pylori* B128 wild type, ΔfapH pflA*, a strain with the kan^R^-*sacB* cassette inserted into *fapH* (Δ*fapH*::*kan*-*sacB* 7), and two isolates with unmarked deletions of *fapH* (designated Δ*fapH* 4 and Δ*fapH* 9) were stab inoculated into soft agar medium that contained 0 or 200 μg/ml bacitracin and the diameters of the resulting swim halos were measured following a 7-d incubation period. Bars indicate mean values for swim halo diameters and the error bars indicate the SEM. The asterisk indicates statistically significant differences in swim halo diameters as determined using a two-sample *t* test (*p*-value <0.0001). At least 5 replicates were done for each sample. (**B** and **C**) Growth and motility of *H*. *pylori* B128 wild type (WT), ΔfapH pflA*, Δ*fapH*::*kan*-*sacB* 7, and Δ*fapH* 9 in soft agar medium in the absence (**B**) and presence (**C**) of 200 μg/ml of bacitracin.
(TIF)

S4 Fig *In-situ* structures of the wild-type and Δ*fapH pflA*^T^ motors. Medial slices through *in-situ* structures of *H*. *pylori* B128 wild type (**A**) WT and (**B**) Δ*fapH pflA*^*T*^ motors determined by subtomogram averaging of 786 and 640 particles for wild type and Δ*fapH pflA*^T^ motors, respectively. Electron densities corresponding to periplasmic accessory structures are absent in the Δ*fapH pflA*^*T*^ motor. Local refinement variation results in variable appearance of the motors. Basal disk (BD), outer disc (OD), cage, outer membrane (OM), and inner membrane (IM) structures are labeled.
(TIF)

S5 Fig AlphaFold modeling of FapH. (**A**) Ribbon diagram of predicted FapH structure predicted by AlphaFold 2 [28]. The N-terminal signal peptide is included in the structure. (**B**) Charge distribution of FapH. Regions of the protein that are dominated by acidic amino acid residues are indicated in red, while regions dominated by basic amino acid residues are indicated in blue. (**C**) Fitting of predicted FapH structure on subunits of FapH ring. The base of the flagellar sheath is shown in green. OD–outer disk.
(TIF)

S6 Fig Assessment of protein-protein interactions in BACTH system on MacConkey-maltose agar. The strains on these plates are the ones that are indicated in **Fig 5**, which shows the results of the β-galactosidase assays for these strains. Strain descriptions are in **S5 Table**. Each plate included a positive control (Pos), which was the strain bearing the plasmids that expressed the T25-zip and T18-zip fusion proteins. Each plate also included a negative control (E229), which was the strain bearing the pKT25 and pUT18 BACTH vectors. (**A**) FapH-FlgP interactions. Strain E233 is a negative control that carries pUT18 and a plasmid expressing the FapH-T25 fusion protein. Strain E173 expresses the T25-FapH and T18-FlgP fusion proteins, strain E172 expresses the FapH-T25 and FlgP-T18 fusion proteins, and strain E171 expresses the FapH-T25 and T18-FlgP fusion proteins. (**B**) FapH-HP1456 interactions. Strain E236 is a negative control that carries pKT25 and a plasmid expressing the T18-HP1456 fusion protein. Strain E185 express the FapH-T18 and HP1456-T25 fusion proteins, and strain E179 expresses the FapH-T25 and T18-HP1456 fusion proteins. (**C**) FlgP-HP1456 interactions. Strain E234 is a negative control that carries pUT18C and a plasmid expressing the FlgP-T25 fusion protein. Strain E193 expresses the T18-HP1456 and T25-FlgP fusion proteins, strain E192 expresses the T18-HP1456 and FlgP-T25 fusion proteins, and strain E188 expresses the HP1456-T25 and T18-FlgP fusion proteins. (**D**) FlgP-HP1454 interactions. Strain E235 is a negative control that carries pUT18 and a plasmid expressing the HP1454-T25 fusion protein. Strain E219 expresses the FlgP-T18 and T25-HP144 fusion proteins, strain E218 expresses the FlgP-T18 and HP1454-T25 fusion proteins, and strain E216 expresses the T18-FlgP and HP1454-T25 fusion proteins. (**E**) FapH-HP1454 interactions. Strain E204 expresses the FapH-T25 and HP1454-T18 fusion proteins, and strain E196 expresses the FapH-T25 and T18-HP1454 fusion proteins. (**F**) FlgP-FlgP interactions. Strain E234 is a negative control that carries pUT18C and a plasmid expressing the FlgP-T25 fusion protein. Strain E224 expressed the T25-FlgP and FlgP-T18 fusion proteins, strain E223 expresses the T25-FlgP and T18-FlgP fusion proteins, and strain E222 expresses the FlgP-T25 and FlgP-T18 fusion proteins.
(TIF)

S7 Fig Characterization of *H*. *pylori* Δ*hp1456* mutant. (**A**) TEM of a *H*. *pylori* Δ*hp1456* mutant cell. (**B**) Cells were visualized by TEM and the number of flagella per cell were counted for each strain (n = 80 for wild type, n = 129 for Δ*hp1456* mutant). An ANOVA analysis of the data indicated there was no significant difference between the strains in the number of flagella per cell. (**C**) *H*. *pylori* Δ*hp1456* mutant and B128 wild type were stab inoculated into soft agar medium that contained no bacitracin or 200 μg/ml bacitracin (+ bac) and the diameters of the resulting swim halos were measured following a 7-d incubation period. Bars indicate mean values for swim halo diameters. The average swim halo diameters for the two strains in the absence or presence of bacitracin were not significantly different as determined using a two-sample *t* test. At least 5 replicates were done for each sample. (**D**) Efficiency of plating assays with *H*. *pylori* Δ*hp1456* mutant and B128 wild type on TSA-HS (-bac) and TSA-HS supplemented with 200 μg/ml bacitracin (+bac). Cells from freshly grown cultures of the strains were resuspended in tryptic soy broth to the same cell densities. Ten-fold serial dilutions of the resuspensions (10^0^ to 10^−8^) were then spotted onto the media, and the cultures were incubated for 7 d. (**E** and **F**) Medial sliced view of subtomogram averaged *in-situ* structures of flagellar motors from Δ*hp1456* mutant (**E**) and wild-type *H*. *pylori* B128 (**F**). OD–outer disk, BD–basal disk, OM- outer membrane, IM–inner membrane.
(TIF)

S8 Fig Construction of the *H*. *pylori* B128 Δ*fapH* mutant. **(A** and **B)** Construction of an unmarked deletion in *hp0838*. In strain H6, *hp0838* has been replaced with the kan^R^-*sacB* cassette (**A**), which was replaced with the unmarked deletion of *hp0838* to generate strain H16 (**B**). **(C)** The arrow indicates the predicted 4-kbp PCR product in lane 2 resulting from amplification of region around *hp0838* using gDNA from strain H6 and primers 59 and 62. Goldbio 1kbp DNA ladder is shown in the lane 1. **(D)** Arrows indicate PCR products of expected sizes resulting from amplification of region around *hp0838* in strains H16 (lane 2; expected size 1 kbp) and H2 (lane 3; expected size 1.5 kbp) using primers 59 and 62. Goldbio 1kb DNA ladder is shown in lane 1. **(E)** PCR with H122 (lane 1) using primers 169 and 171. NEB 1kDa DNA ladder. 618 bp band (H122). gDNA PCR products were confirmed by sequencing (Eton Biosciences).
(TIF)

S9 Fig DNA sequences of the insertions in plasmid pHel3 to generate plasmids pHel3-GG and pHel3-myc. (**A**) The DNA sequence was synthesized and inserted into the BamHI and XhoI sites of the *H*. *pylori* shuttle vector pHel3 [51] by Azenta Life Sciences to create plasmid pHel3-GG. Sequence in red contains the predicted promoter for *fliF* in *H*. *pylori* 26695. Sequence between NheI and NcoI sites is the Shine-Dalgarno sequence from *H*. *pylori* 26695 *ureA*. Tandem BspQ1 sites are indicated in blue, with the underlined sequence corresponding to the BspQ1 site on the top strand and the italicized sequence corresponding to the BspQ1 site on the bottom strand. Start codon for the cloned gene of interest is within the NcoI site and the stop codon is indicated in green. The sequence introduces a unique NheI site that can be used in conjunction with the unique BamHI for switching the promoter in the vector. (**B**) The DNA sequence was synthesized and inserted into the BamHI and XhoI sites of the *H*. *pylori* shuttle vector pHel3 by Azenta Life Sciences to create plasmid pHel3-myc. In addition to the features described for pHel3-GG, the synthetic DNA introduced a coding sequence for a flexible glycine- and serine-rich linker [65], c-myc epitope, and a DDDDK epitope between the tandem BspQI sites (indicated in blue) and stop codon (indicated in green). (**C**) Nucleotide and amino acid sequences for flexible linker (amino acid sequence in purple), c-myc epitope (amino acid sequence in red), and DDDDK epitope (in blue).
(TIF)

S1 Table Homologs of FapH and FlgP in *Helicobacter* species and other members of Campylobacterota. (XLSX)

S2 Table Mutations identified from whole genome sequencing of *fapH* and *hp1456* mutants. (DOCX)

S3 Table Proteins identified from co-immunoprecipitation assays. (XLSX)

S4 Table Strains and plasmids used in study. (XLSX)

S5 Table List of primers used in study. (XLSX)

S6 Table Raw data used to generate graphs. (XLSX)
