# Correction: A *Helicobacter pylori* flagellar motor accessory is needed to maintain the barrier function of the outer membrane during flagellar rotation

**DOI:** 10.1371/journal.ppat.1013912

**Published:** 2026-02-02

**Authors:** Kyle Rosinke, Shoichi Tachiyama, Jan Mrázek, Jun Liu, Timothy R. Hoover

The third author's name is spelled incorrectly. The correct name is: Jan Mrázek. The correct citation is: Rosinke K, Tachiyama S, Mrázek J, Liu J, Hoover TR (2025) A Helicobacter pylori flagellar motor accessory is needed to maintain the barrier function of the outer membrane during flagellar rotation. PLoS Pathog 21(1):e1012860. https://doi.org/10.1371/journal.ppat.1012860.
