# Supplementary figures and images for: A Helicobacter pylori flagellar motor accessory is needed to maintain the barrier function of the outer membrane during flagellar rotation
Source: PLoS Pathog. 2025 Jan 10;21(1):e1012860. doi: 10.1371/journal.ppat.1012860 (PMC11756786; doi:10.1371/journal.ppat.1012860)

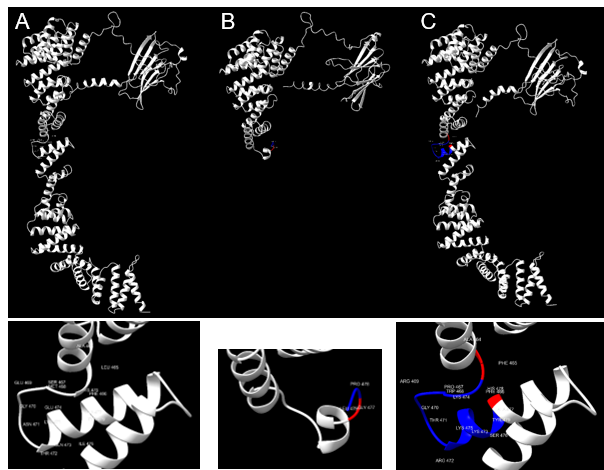

Supplement: S1 Fig — Predicted structures generated by AlphaFold 2 [28] are shown for (A) native PflA, (B) truncated PflA expressed in ΔfapH pflAT, and (C) PflA* variant expressed in ΔfapH pflA*. Close ups of the regions where the PflA proteins differ are shown below each modeled structure. Red coloring indicates residues where mutations occurred and blue coloring indicates resides that are changed downstream of where the mutations occur. (TIF) [file ppat.1012860.s001.tif]

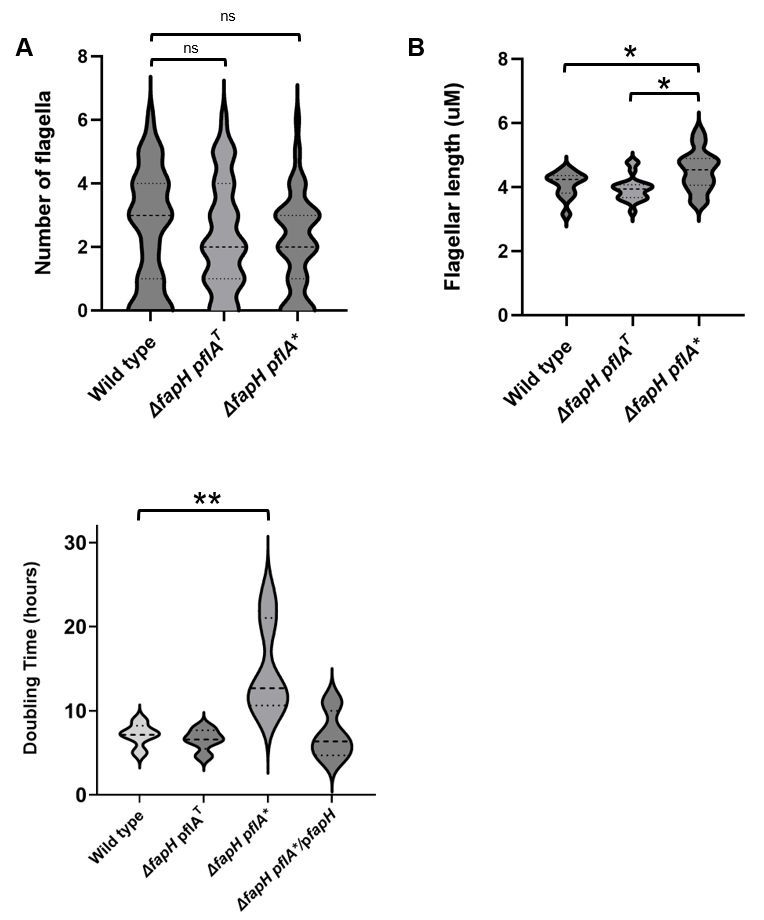

Supplement: S2 Fig — (A) Number of flagella per cell for H. pylori B128 wild type, ΔfapH pflAT, and ΔfapH pflA*. Cells were grown in liquid media, harvested, and visualized by TEM and the number of flagella per cell (n = 100) were counted for each strain. An ANOVA analysis of the data indicated there was no significant difference between the strains in the number of flagella per cell. ns–not significant. (B) Lengths of the flagellar filaments were measured using ImageJ and an ANOVA analysis of the data was done to assess the statistical significance of any differences. The asterisk (*) indicates a p-value < 0.005. (C) Growth rates of H. pylori strains in brain heart infusion (BHI) supplemented with 5% heat-inactivated horse serum were determined by measuring OD600 values of the cultures at various times. Calculated doubling times for the strains were: wild type B128–7.1 h; ΔfapH pflAT—6.6 h; ΔfapH pflA* - 13.1 h; ΔfapH pflA*/ pfapH—7.0 h. An ANOVA analysis of the data was done to assess the statistical significance of any differences. The double asterisk (**) indicates a p-value < 0.05. (TIF) [file ppat.1012860.s002.tif]

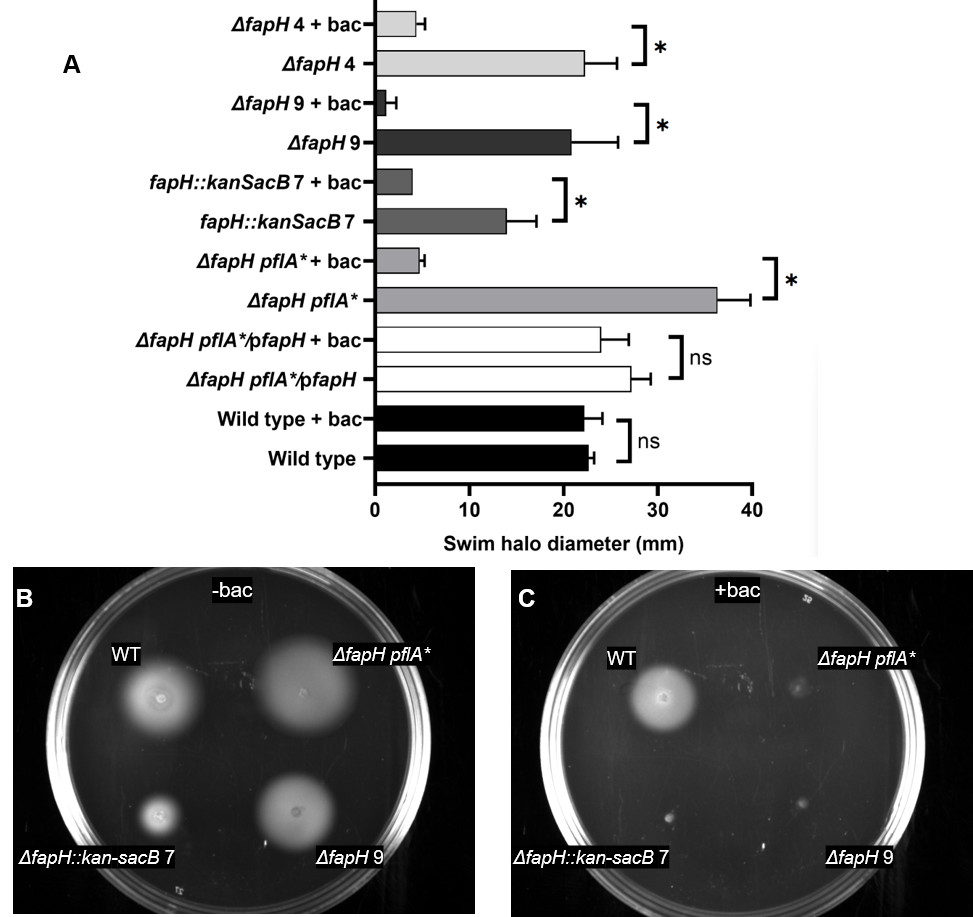

Supplement: S3 Fig — (A) H. pylori B128 wild type, ΔfapH pflA*, a strain with the kanR-sacB cassette inserted into fapH (ΔfapH::kan-sacB 7), and two isolates with unmarked deletions of fapH (designated ΔfapH 4 and ΔfapH 9) were stab inoculated into soft agar medium that contained 0 or 200 μg/ml bacitracin and the diameters of the resulting swim halos were measured following a 7-d incubation period. Bars indicate mean values for swim halo diameters and the error bars indicate the SEM. The asterisk indicates statistically significant differences in swim halo diameters as determined using a two-sample t test (p-value <0.0001). At least 5 replicates were done for each sample. (B and C) Growth and motility of H. pylori B128 wild type (WT), ΔfapH pflA*, ΔfapH::kan-sacB 7, and ΔfapH 9 in soft agar medium in the absence (B) and presence (C) of 200 μg/ml of bacitracin. (TIF) [file ppat.1012860.s003.tif]

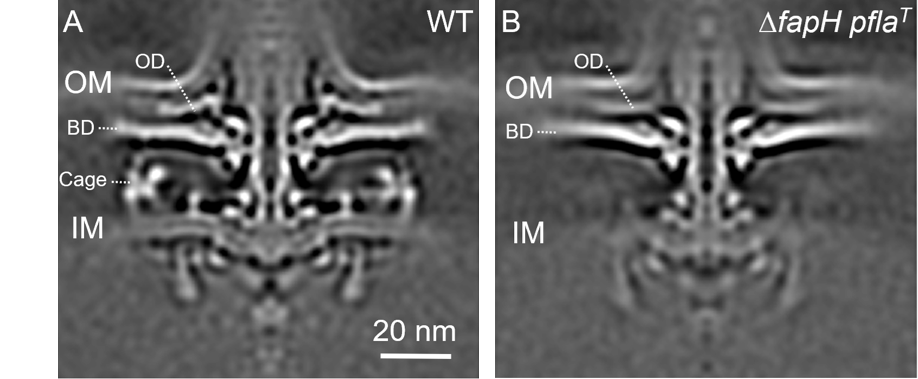

Supplement: S4 Fig — Medial slices through in-situ structures of H. pylori B128 wild type (A) WT and (B) ΔfapH pflAT motors determined by subtomogram averaging of 786 and 640 particles for wild type and ΔfapH pflAT motors, respectively. Electron densities corresponding to periplasmic accessory structures are absent in the ΔfapH pflAT motor. Local refinement variation results in variable appearance of the motors. Basal disk (BD), outer disc (OD), cage, outer membrane (OM), and inner membrane (IM) structures are labeled. (TIF) [file ppat.1012860.s004.tif]

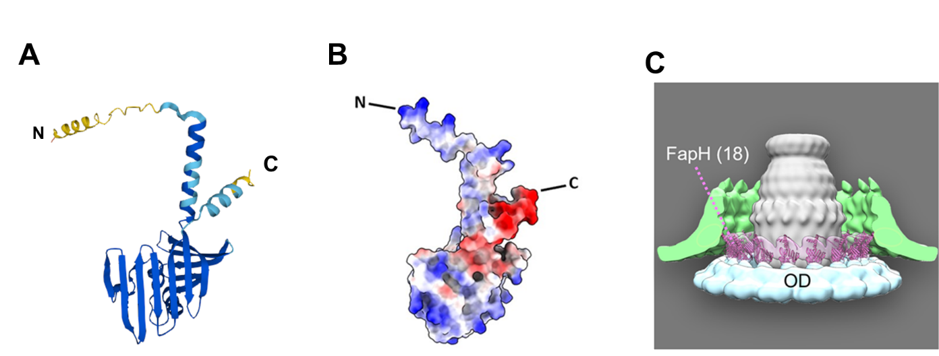

Supplement: S5 Fig — (A) Ribbon diagram of predicted FapH structure predicted by AlphaFold 2 [28]. The N-terminal signal peptide is included in the structure. (B) Charge distribution of FapH. Regions of the protein that are dominated by acidic amino acid residues are indicated in red, while regions dominated by basic amino acid residues are indicated in blue. (C) Fitting of predicted FapH structure on subunits of FapH ring. The base of the flagellar sheath is shown in green. OD–outer disk. (TIF) [file ppat.1012860.s005.tif]

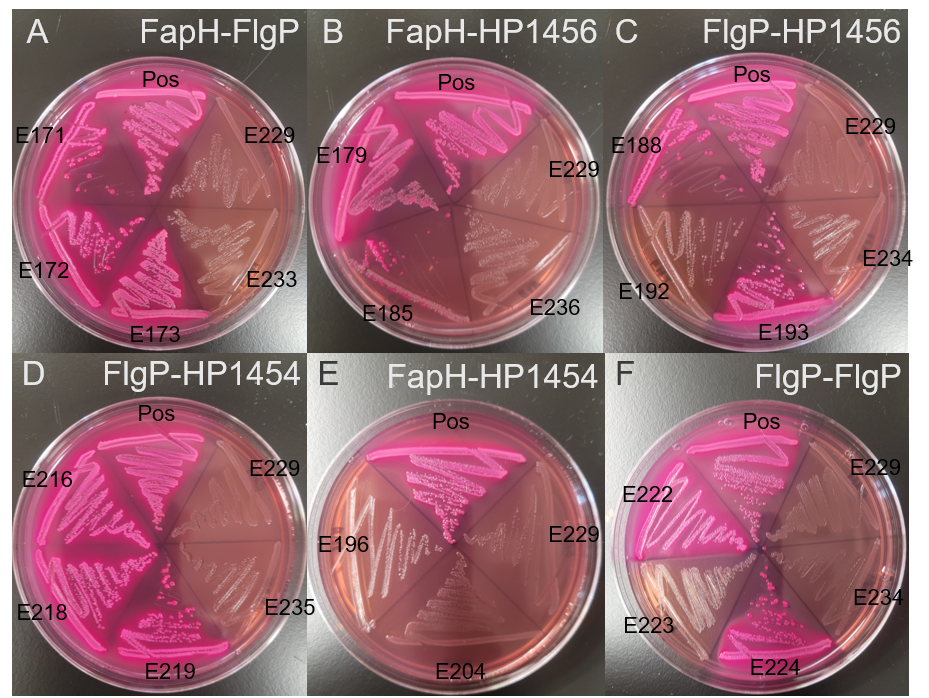

Supplement: S6 Fig — The strains on these plates are the ones that are indicated in Fig 5, which shows the results of the β-galactosidase assays for these strains. Strain descriptions are in S5 Table. Each plate included a positive control (Pos), which was the strain bearing the plasmids that expressed the T25-zip and T18-zip fusion proteins. Each plate also included a negative control (E229), which was the strain bearing the pKT25 and pUT18 BACTH vectors. (A) FapH-FlgP interactions. Strain E233 is a negative control that carries pUT18 and a plasmid expressing the FapH-T25 fusion protein. Strain E173 expresses the T25-FapH and T18-FlgP fusion proteins, strain E172 expresses the FapH-T25 and FlgP-T18 fusion proteins, and strain E171 expresses the FapH-T25 and T18-FlgP fusion proteins. (B) FapH-HP1456 interactions. Strain E236 is a negative control that carries pKT25 and a plasmid expressing the T18-HP1456 fusion protein. Strain E185 express the FapH-T18 and HP1456-T25 fusion proteins, and strain E179 expresses the FapH-T25 and T18-HP1456 fusion proteins. (C) FlgP-HP1456 interactions. Strain E234 is a negative control that carries pUT18C and a plasmid expressing the FlgP-T25 fusion protein. Strain E193 expresses the T18-HP1456 and T25-FlgP fusion proteins, strain E192 expresses the T18-HP1456 and FlgP-T25 fusion proteins, and strain E188 expresses the HP1456-T25 and T18-FlgP fusion proteins. (D) FlgP-HP1454 interactions. Strain E235 is a negative control that carries pUT18 and a plasmid expressing the HP1454-T25 fusion protein. Strain E219 expresses the FlgP-T18 and T25-HP144 fusion proteins, strain E218 expresses the FlgP-T18 and HP1454-T25 fusion proteins, and strain E216 expresses the T18-FlgP and HP1454-T25 fusion proteins. (E) FapH-HP1454 interactions. Strain E204 expresses the FapH-T25 and HP1454-T18 fusion proteins, and strain E196 expresses the FapH-T25 and T18-HP1454 fusion proteins. (F) FlgP-FlgP interactions. Strain E234 is a negative control that carries pUT18C and a plasmid e [file ppat.1012860.s006.tif]

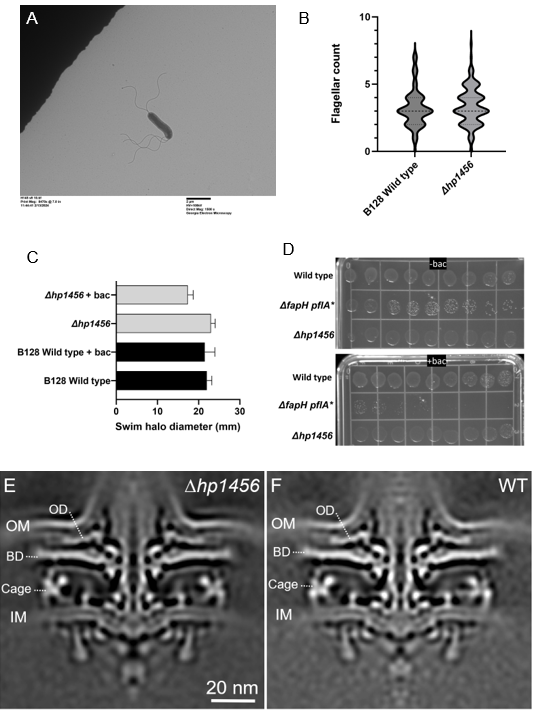

Supplement: S7 Fig — (A) TEM of a H. pylori Δhp1456 mutant cell. (B) Cells were visualized by TEM and the number of flagella per cell were counted for each strain (n = 80 for wild type, n = 129 for Δhp1456 mutant). An ANOVA analysis of the data indicated there was no significant difference between the strains in the number of flagella per cell. (C) H. pylori Δhp1456 mutant and B128 wild type were stab inoculated into soft agar medium that contained no bacitracin or 200 μg/ml bacitracin (+ bac) and the diameters of the resulting swim halos were measured following a 7-d incubation period. Bars indicate mean values for swim halo diameters. The average swim halo diameters for the two strains in the absence or presence of bacitracin were not significantly different as determined using a two-sample t test. At least 5 replicates were done for each sample. (D) Efficiency of plating assays with H. pylori Δhp1456 mutant and B128 wild type on TSA-HS (-bac) and TSA-HS supplemented with 200 μg/ml bacitracin (+bac). Cells from freshly grown cultures of the strains were resuspended in tryptic soy broth to the same cell densities. Ten-fold serial dilutions of the resuspensions (100 to 10−8) were then spotted onto the media, and the cultures were incubated for 7 d. (E and F) Medial sliced view of subtomogram averaged in-situ structures of flagellar motors from Δhp1456 mutant (E) and wild-type H. pylori B128 (F). OD–outer disk, BD–basal disk, OM- outer membrane, IM–inner membrane. (TIF) [file ppat.1012860.s007.tif]

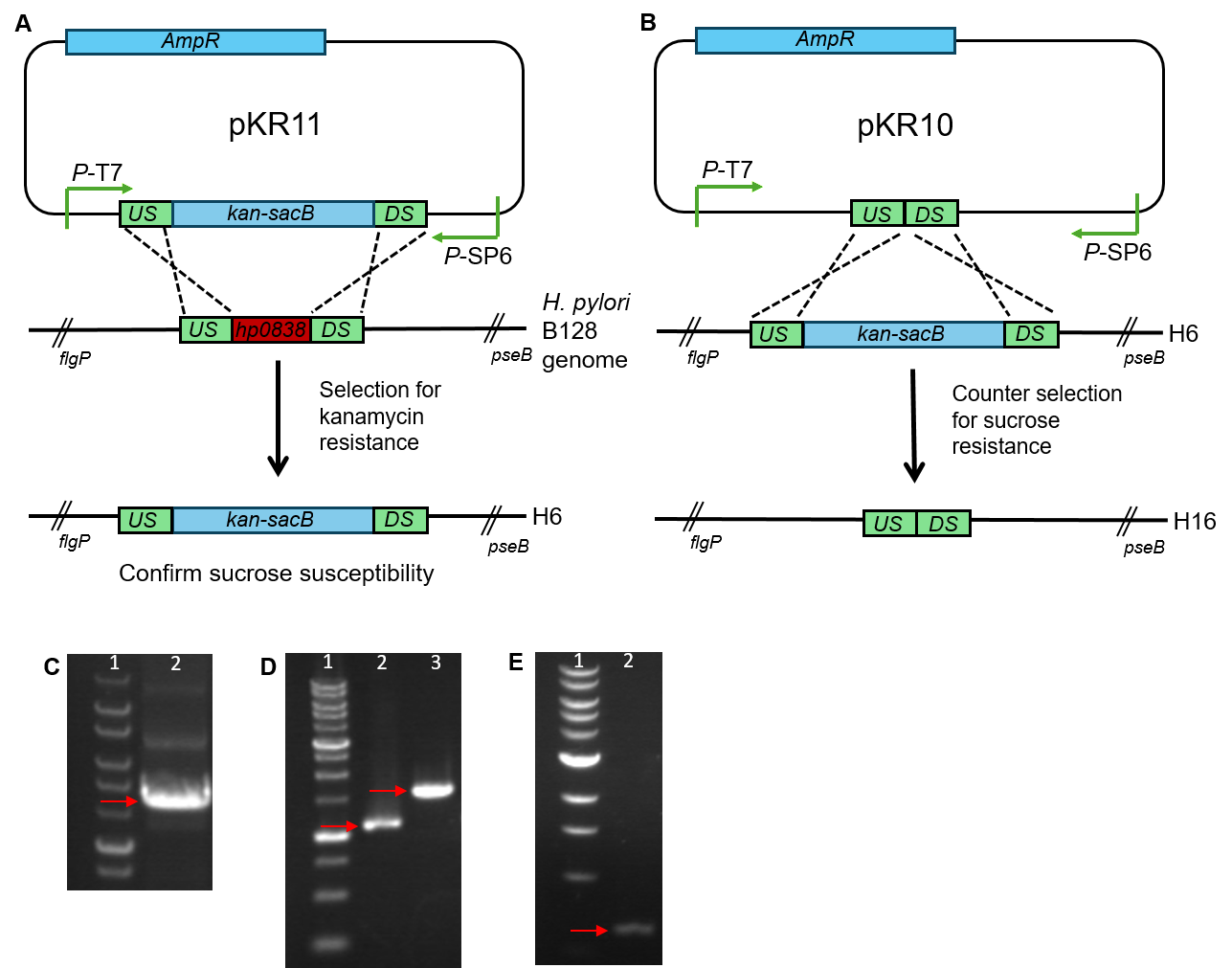

Supplement: S8 Fig — (A and B) Construction of an unmarked deletion in hp0838. In strain H6, hp0838 has been replaced with the kanR-sacB cassette (A), which was replaced with the unmarked deletion of hp0838 to generate strain H16 (B). (C) The arrow indicates the predicted 4-kbp PCR product in lane 2 resulting from amplification of region around hp0838 using gDNA from strain H6 and primers 59 and 62. Goldbio 1kbp DNA ladder is shown in the lane 1. (D) Arrows indicate PCR products of expected sizes resulting from amplification of region around hp0838 in strains H16 (lane 2; expected size 1 kbp) and H2 (lane 3; expected size 1.5 kbp) using primers 59 and 62. Goldbio 1kb DNA ladder is shown in lane 1. (E) PCR with H122 (lane 1) using primers 169 and 171. NEB 1kDa DNA ladder. 618 bp band (H122). gDNA PCR products were confirmed by sequencing (Eton Biosciences). (TIF) [file ppat.1012860.s008.tif]

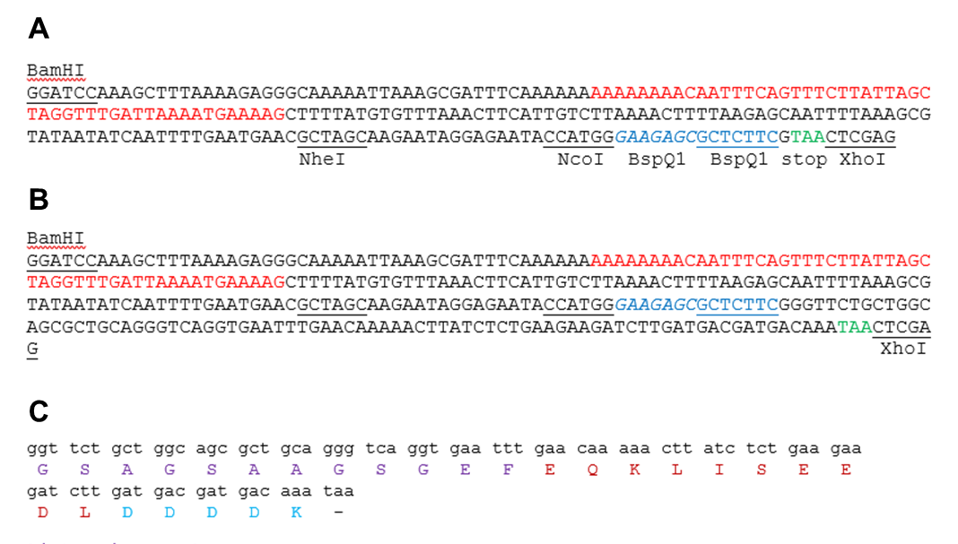

Supplement: S9 Fig — (A) The DNA sequence was synthesized and inserted into the BamHI and XhoI sites of the H. pylori shuttle vector pHel3 [51] by Azenta Life Sciences to create plasmid pHel3-GG. Sequence in red contains the predicted promoter for fliF in H. pylori 26695. Sequence between NheI and NcoI sites is the Shine-Dalgarno sequence from H. pylori 26695 ureA. Tandem BspQ1 sites are indicated in blue, with the underlined sequence corresponding to the BspQ1 site on the top strand and the italicized sequence corresponding to the BspQ1 site on the bottom strand. Start codon for the cloned gene of interest is within the NcoI site and the stop codon is indicated in green. The sequence introduces a unique NheI site that can be used in conjunction with the unique BamHI for switching the promoter in the vector. (B) The DNA sequence was synthesized and inserted into the BamHI and XhoI sites of the H. pylori shuttle vector pHel3 by Azenta Life Sciences to create plasmid pHel3-myc. In addition to the features described for pHel3-GG, the synthetic DNA introduced a coding sequence for a flexible glycine- and serine-rich linker [65], c-myc epitope, and a DDDDK epitope between the tandem BspQI sites (indicated in blue) and stop codon (indicated in green). (C) Nucleotide and amino acid sequences for flexible linker (amino acid sequence in purple), c-myc epitope (amino acid sequence in red), and DDDDK epitope (in blue). (TIF) [file ppat.1012860.s009.tif]
