# Supplementary material for: A Helicobacter pylori flagellar motor accessory is needed to maintain the barrier function of the outer membrane during flagellar rotation
Source: PLoS Pathog. 2025 Jan 10;21(1):e1012860. doi: 10.1371/journal.ppat.1012860 (PMC11756786; doi:10.1371/journal.ppat.1012860)
Supplement: S2 Table — (DOCX) [file ppat.1012860.s011.docx]

**S2 Table**. **Mutations identified from whole genome sequencing of *fapH* and *hp1456* mutants.**

| **Δ*fapH* *pflA*^T^ (lab strain H16)** | | | | | |
| --- | --- | --- | --- | --- | --- |
| **NCBI designation** | **26695 locus tag** | **Gene/ description** | **Mutation** | ***^a^*Impact** | ***^b^*Frequency** |
| *CV725_RS00010* | HP1527 | *comH*, competence protein | Δ1 bp (708/1440 nt) | Val236fs | 95% |
| *CV725_RS00960* | HP1274 | *pflA*, paralyzed flagella protein | +G (1428/2406 nt) | Ala477fs | 92.9% |
| *CV725_RS02305CV725_RS02315* | HP0427  23S rRNA | US of *hp0427* (hypothetical protein), US of 23S rRNA gene | +GTTGGATGATTGGATG intergenic (‑234/‑736) | unknown | 97.4% |
| *mreC* | HP1372 | *mreC*, rod shape-determining protein | codon-220 (TAC→CAC) | Tyr220His | 97.7% |
| *CV725_RS02745* | HP0098 | *thrC*, threonine synthase | codon-417 (GAA→AAA) | Glu417Lys | 96.3% |
| *CV725_RS04505* | HP0475 | *modD*, molybdenum ABC transport protein | codon-155 (TTA→TTG) | Leu155Leu | 96% |
| *CV725_RS04785* | HP0838 | *fapH* | codon-18 (GTC→GCC)  Δ515 bp (69-584/618 nt) | Val18Ala  deletion | 84.1% |
| *CV725_RS05360CV725_RS05365* | HP0726  HP0725 | DS of *hp0726* outer membrane protein, US of *hopP* | (T)_20→19_ intergenic (+121/‑173) | unknown | 84.6% |
| *CV725_RS06350* | HP0527 | cag pathogenicity island protein (cag7) | codon-936  (ACG→ACC)  codon-752  (ACC→ACG)  codon-742  (GCT→GCC) | Thr936Thr  Thr752Thr  Ala742Ala | 95.6%  97.8%  98.5% |
| *CV725_RS06500* | HP0499 | *pldA*, phospholipase A pseudogene | (G)_10→11_  (683/1070 nt) | phase ‘off’ to phase ’on’ | 67.1% |
| *babA*  *CV725_RS06645* | HP1243  tRNA_fMet | US of *babA*, DS of tRNA_fMet | (T)_13→12_  intergenic (‑131/+149) | unknown | 91.2% |
| *CV725_RS08550*  *queF* | HP1412  HP1413 | DS of *hp1412* (hypothetical protein), DS of *queF* | (C)_11→10_  intergenic (+11/+121) | unknown | 96.8% |
|  |  |  |  |  |  |
| **Δ*fapH* *pflA**** **(lab strain H54, parental strain H16)** | | | | | |
| *CV725_RS00010* | HP1527 | *comH*, competence protein | Δ1 bp (708/1440 nt) | Val236fs | 98.8% |
| ********CV725_RS00395* | HP1451 | spoIIIJ-associated protein | +T (689/798 nt) | Tyr230fs | 98.1% |
| *CV725_RS00960* | HP1274 | *pflA*, paralyzed flagella protein | +G (1428/2406 nt)  Δ1 bp (1391/2406 nt) | offsetting fs mutations | 98.4%  98.5% |
| *maf*  *CV725_RS01150* | HP1239  HP1240 | DS of *maf*, US of *hp1239* (hypothetical protein) | (A)_13→14_  intergenic (+61/+394) | unknown | 80.5% |
| *mreC* | HP1372 | *mreC*, rod shape-determining protein | codon-220 (TAC→CAC) | Tyr220His | 98.8% |
| *CV725_RS02745* | HP0098 | *thrC*, threonine synthase | codon-417 (GAA→AAA) | Glu417Lys | 98.9% |
| ********tlpB* | HP0103 | *tlpB,* methyl-accepting chemotaxis protein | codon-26  (GGG→GAG) | Gly26Glu | 98.6% |
| ********CV725_RS03745* | HP0298 | *dppA*, dipeptide ABC transporter, periplasmic dipeptide-binding protein | (T)_10→9_  coding (30/1644 nt) | Phe10fs | 96.4% |
| ********CV725_RS04335* | HP1028 | hypothetical protein | codon-16  (GCT→GTT) | Ala16Val | 98.5% |
| *CV725_RS04505* | HP0475 | *modD*, molybdenum ABC transport protein | codon-155 (TTA→TTG) | Leu155Leu | 99.1% |
| *CV725_RS04600CV725_RS04605* | HP0875  HP0876 | US of *katA,* US of *frpB* | (A)_5→6_ intergenic (‑34/‑290) | unknown | 83.4% |
| *CV725_RS04785* | HP0838 | *fapH* | codon-18 (GTC→GCC)  Δ515 bp (69-584/618 nt) | Val18Ala  deletion | 99.1% |
| *CV725_RS06350* | HP0527 | cag pathogenicity island protein (cag7) | codon-752  (ACC→ACG)  codon-742  (GCT→GCC) | Thr752Thr  Ala742Ala | 96.2%  99.3% |
| *CV725_RS06500* | HP0499 | *pldA*, phospholipase A pseudogene | (G)_10→11_  (683/1070 nt) | phase ‘off’ to phase ’on’ | 62.8% |
| *babA*  *CV725_RS06645* | HP1243  tRNA_fMet | US of *babA*, DS of tRNA_fMet | (T)_13→12_  intergenic (‑131/+149) | unknown | 86.2% |
| *CV725_RS08550*  *queF* | HP1412  HP1413 | DS of *hp1412* (hypothetical protein), DS of *queF* | (C)_11→10_  intergenic (+11/+121) | unknown | 94.6% |
|  |  |  |  |  |  |
| ***fapH::kan-sacB*** **(lab strain H180-7)** | | | | | |
| *CV725_RS00720*  *CV725_RS00725* | HP1322  HP1321 | DS of HP1321 conserved hypothetical ATP-binding protein and US of HP1322 hypothetical protein. | (ATACATAA)_10→5_  intergenic (‑194/+7) | unknown | 99.7% |
| *CV725_RS02305CV725_RS02315* | HP0427  23S rRNA | US of *hp0427* (hypothetical protein), US of 23S rRNA gene | +32 bp intergenic (‑234/‑736) | unknown | 90.5% |
| *CV725_RS02465* | HP1375 | N‑6 DNA methylase | (C)_14→13_  pseudogene (3224/3372 nt) | restore full-length ORF | 88.3% |
| *CV725_RS04600* *CV725_RS04605* | HP0875  HP0876 | US of HP0875 *katA,* US of HP0876 *frpB* | (A)_5→6_ intergenic (‑34/‑290) | unknown | 94.4% |
| *CV725_RS04785* | HP0838 | *fapH* | codon-18 (GAT→GAC)  Δ515 bp (69-584/618 nt) | Asp201Asp  deletion | 100.0% |
| *CV725_RS06350* | HP0527 | cag pathogenicity island protein (cag7) | codon-752  (ACC→ACG)  codon-742  (GCT→GCC) | Thr752Thr  Ala742Ala | 87.5%  82.1% |
| *CV725_RS06500* | HP0499 | *pldA*, phospholipase A pseudogene | (G)_10→9_  (683/1070 nt) | phase ‘off’ to phase ’off’ | 93.7% |
| *CV725_RS07515* *queF* | HP1412  HP1413 | DS of HP1412 hypothetical protein and DS of queF | (C)_11→10_  intergenic (+11/+121) | unknown | 96.0% |
|  |  |  |  |  |  |
| **Δ*fapH* (lab strain H181-4, parental strain H180-7)** | | | | | |
| *CV725_RS00720*  *CV725_RS00725* | HP1322  HP1321 | DS of HP1321 conserved hypothetical ATP-binding protein and US of HP1322 hypothetical protein. | (ATACATAA)_10→5_  intergenic (‑194/+7) | unknown | 99.6% |
| *CV725_RS02305CV725_RS02315* | HP0427  23S rRNA | US of *hp0427* (hypothetical protein), US of 23S rRNA gene | A→G intergenic (‑235/‑735) | unknown | 86.2% |
| *CV725_RS02465* | HP1375 | N‑6 DNA methylase | (C)_14→13_  pseudogene (3224/3372 nt) | restore full-length ORF | 92.3% |
| *CV725_RS03080* | HP0164 | *arsS*, histidine kinase | (C)_11→12_  coding (1261/1281 nt) | K^252^-Ile^253^-stop to E^252^-Asp^253^-Leu^254^-stop | 80.8% |
| *CV725_RS04600* *CV725_RS04605* | HP0875  HP0876 | US of HP0875 *katA,* US of HP0876 *frpB* | (A)_5→6_ intergenic (‑34/‑290) | unknown | 92.8% |
| *CV725_RS04785* | HP0838 | *fapH* | codon-18 (GTC→GCC)  codon-201  (GAT→GAC)  Δ515 bp (69-584/618 nt) | Val18Ala  Asp201Asp  deletion | 100%  99.0%  100% |
| *CV725_RS06350* | HP0527 | cag pathogenicity island protein (cag7) | codon-742  (GCT→GCC) | Ala742Ala | 82.3% |
| *CV725_RS06500* | HP0499 | *pldA*, phospholipase A pseudogene | (G)_10→9_  (683/1070 nt) | phase ‘off’ to phase ’off’ | 93.3% |
| *CV725_RS07515* *queF* | HP1412  HP1413 | DS of HP1412 hypothetical protein and DS of queF | (C)_11→10_  intergenic (+11/+121) | unknown | 98.0% |
|  |  |  |  |  |  |
| **Δ*fapH* (lab strain H181-9, parental strain H180-7)** | | | | | |
| *CV725_RS00720*  *CV725_RS00725* | HP1322  HP1321 | DS of HP1321 conserved hypothetical ATP-binding protein and US of HP1322 hypothetical protein. | (ATACATAA)_10→5_  intergenic (‑194/+7) | unknown | 100% |
| *CV725_RS02305CV725_RS02315* | HP0427  23S rRNA | US of *hp0427* (hypothetical protein), US of 23S rRNA gene | +32 bp intergenic (‑234/‑736) | unknown | 89.3% |
| *CV725_RS02465* | HP1375 | N‑6 DNA methylase | (C)_14→13_  pseudogene (3224/3372 nt) | restore full-length ORF | 84.3% |
| *CV725_RS04600* *CV725_RS04605* | HP0875  HP0876 | US of HP0875 *katA,* US of HP0876 *frpB* | (A)_5→6_ intergenic (‑34/‑290) | unknown | 97.1% |
| CV725_RS04780 | HP0839 | outer membrane protein P1 (ompP1) | +30bp  coding (494‑523/1764 nt | insertion | 100% |
| *CV725_RS04785* | HP0838 | *fapH* | codon-18 (GTC→GCC)  codon-201  (GAT→GAC)  Δ515 bp (69-584/618 nt) | Val18Ala  Asp201Asp  deletion | 100%  99.1%  100% |
| *CV725_RS06350* | HP0527 | cag pathogenicity island protein (cag7) | codon-752  (ACC→ACG) | Thr752Thr | 82.6% |
| *CV725_RS06500* | HP0499 | *pldA*, phospholipase A pseudogene | (G)_10→9_  (683/1070 nt) | phase ‘off’ to phase ’off’ | 95.2% |
| *CV725_RS07515* *queF* | HP1412  HP1413 | DS of HP1412 hypothetical protein and DS of queF | (C)_11→10_  intergenic (+11/+121) | unknown | 98% |
| *CV725_RS07950* | HP1562 | iron (III) ABC transporter, periplasmic iron-binding protein (ceuE) | Codon-180  (GAG→GAT) | Glu180Asp | 100% |
|  |  |  |  |  |  |
| **Δ*hp1456* (lab strain H145-9)** | | | | | |
| *CV725_RS00365* | HP1465 | LPP20 family lipoprotein | 455 bp→25 bp coding (48‑502/528 nt) | gene deletion | 100% |
| *CV725_RS00720*  *CV725_RS00725* | HP1322  HP1321 | DS of HP1321 conserved hypothetical ATP-binding protein and US of HP1322 hypothetical protein. | (ATACATAA)_10→5_  intergenic (‑194/+7) | unknown | 98.3% |
| *babA* | HP1243 | *babA* | Coding-718  (AGA→CGC)  Coding-719  (AGA→AGG)  Coding-720  (CTC→CTT)  Coding-727  (TAC→TAT) | Arg718Arg  Arg719Arg  Leu720Leu  Tyr727Tyr | 100%  100%  100%  98.6% |
| *CV725_RS02465* | HP1375 | N‑6 DNA methylase | (C)_14→13_  pseudogene (3224/3372 nt) | restore full-length ORF | 91.8% |
| *hcpE* | HP0235 | Sel1‑like repeat protein HcpE | +CAGGGTGTTTT  coding (384/1068 nt) | Frameshift, out of frame | 93.7% |
| *pyrG*  *CV725_RS03950* | HP0349  HP0350 | US of HP0349 *pyrG,* US of HP0350 hypothetical protein | (A)17→16  intergenic (‑94/‑161) | unknown | 91.9% |
| *CV725_RS04600* *CV725_RS04605* | HP0875  HP0876 | US of HP0875 *katA,* US of HP0876 *frpB* | (A)_5→6_ intergenic (‑34/‑290) | unknown | 92.6% |
| *CV725_RS06350* | HP0527 | cag pathogenicity island protein (cag7) | codon-752  (ACC→ACG) | Thr752Thr | 91.5% |
| *CV725_RS06500* | HP0499 | *pldA*, phospholipase A pseudogene | (G) _10→11_  (683/1070 nt) | phase ‘off’ to phase ’on’ | 86.5% |
| *CV725_RS07515* *queF* | HP1412  HP1413 | DS of HP1412 hypothetical protein and DS of queF | (C)_11→10_  intergenic (+11/+121) | unknown | 97.1% |
| CV725_RS07535 | HP1416 | lipopolysaccharide 1,2-glucosyltransferase (rfaJ) | Coding-26  (GGC→GAC) | Gly26Asp | 99.8% |

*^a^*”fs” indicates frameshift

^b^Frequency is the number of times the mutation was identified relative to the total number of reads for that sequence.

*Not found in Δ*fapH* *pflA*^T^.

Displaying mutations that are found in > 80% of reads and any mutations in known flagellar genes.

Secondary mutations in flagellar genes are highlighted in yellow.
